# Comparison of genotyping assays for detection of targeted CRISPR/Cas mutagenesis in highly polyploid sugarcane

**DOI:** 10.3389/fgeed.2024.1505844

**Published:** 2024-12-12

**Authors:** Eleanor J. Brant, David May, Ayman Eid, Fredy Altpeter

**Affiliations:** ^1^ Agronomy Department, Plant Molecular and Cellular Biology Program, Genetics Institute, University of Florida, IFAS-Institute of Food and Agricultural Science, Gainesville, FL, United States; ^2^ DOE Center for Advanced Bioenergy and Bioproducts Innovation, Gainesville, FL, United States

**Keywords:** polyploid, genotyping, mutations, CRISPR/Cas9, high resolution melt, capillary electrophoresis, Cas9 RNP, sugarcane

## Abstract

Sugarcane (*Saccharum* spp.) is an important biofuel feedstock and a leading source of global table sugar. *Saccharum* hybrid cultivars are highly polyploid (2n = 100–130), containing large numbers of functionally redundant hom(e)ologs in their genomes. Genome editing with sequence-specific nucleases holds tremendous promise for sugarcane breeding. However, identification of plants with the desired level of co-editing within a pool of primary transformants can be difficult. While DNA sequencing provides direct evidence of targeted mutagenesis, it is cost-prohibitive as a primary screening method in sugarcane and most other methods of identifying mutant lines have not been optimized for use in highly polyploid species. In this study, non-sequencing methods of mutant screening, including capillary electrophoresis (CE), Cas9 RNP assay, and high-resolution melt analysis (HRMA), were compared to assess their potential for CRISPR/Cas9-mediated mutant screening in sugarcane. These assays were used to analyze sugarcane lines containing mutations at one or more of six sgRNA target sites. All three methods distinguished edited lines from wild type, with co-mutation frequencies ranging from 2% to 100%. Cas9 RNP assays were able to identify mutant sugarcane lines with as low as 3.2% co-mutation frequency, and samples could be scored based on undigested band intensity. CE was highlighted as the most comprehensive assay, delivering precise information on both mutagenesis frequency and indel size to a 1 bp resolution across all six targets. This represents an economical and comprehensive alternative to sequencing-based genotyping methods which could be applied in other polyploid species.

## 1 Introduction

Over the last decade, advances in genome editing technologies have energized crop improvement efforts and gene functional studies. The clustered regularly interspaced short palindromic repeat (CRISPR) genome editing platform has revolutionized plant biotechnology. CRISPR utilizes sequence specific nucleases (SSNs), such as CRISPR-associated protein 9 (Cas9), to create double-stranded DNA breaks. DNA repair is typically carried out through the non-homologous end joining (NHEJ) and homology directed repair (HDR) pathways, resulting in gene knockouts *via* insertions/deletions (indels), or precision editing *via* targeted nucleotide substitutions, respectively (Schmidt et al., 2019). In contrast to other SSN technologies reliant on protein-DNA interactions for target specificity, Cas9 nucleases are directed by short 20 nucleotide single guide RNAs (sgRNAs) complementary to the desired target region. This provides a high level of design flexibility and enables multiplex editing, as sgRNA sites only need to be followed by a 3 bp protospacer adjacent motif (PAM) to be recognized and targeted by the Cas nuclease (Knott and Doudna, 2018).

Polyploid crops are vital contributors to global food, fuel, and forage production, with species such as sugarcane (*Saccharum* spp. Hybrid, 2*n* = 10–13*x* = 100–130), wheat (*Triticum aestivum* L., 2*n* = 6*x* = 42), potato (*Solanum tuberosum* L., 2*n* = 4*x =* 48), sweet potato (*Ipomoea batatas* L., 2*n* = 6*x* = 90), banana (*Musa* spp., 2*n* = 2-3*x* = 22–33), and cotton (*Gossypium hirsutum* L., 2*n* = 4*x* = 52) all ranking in the top 20 for gross production in 2021 (FAO, 2022). While CRISPR/Cas has been applied in polyploid species (May et al., 2023a), adoption of high-throughput, cost-effective methods for mutant screening has remained elusive (Liu et al., 2018; Eid et al., 2021; Kim et al., 2021; May et al., 2023b). As transformation protocols often generate large numbers of transgenic plants, including non-edited lines, genotypic analysis must be undertaken to reveal editing outcomes. In polyploids, the presence of multiple hom(e)ologous gene copies requires quantification of the extent of co-editing to fully assess the phenotypic impacts of targeted genome modifications (Schaart et al., 2021; May et al., 2023a).

While CRISPR holds great potential for multiallelic editing, its rate of success is highly dependent on both sgRNA and target site selection, and the species/cultivar (Liang et al., 2016; Arndell et al., 2019; Ahmad et al., 2023). Within CRISPR edited polyploids, it is common to observe a range of co-mutation frequencies, dependent on the number of copies/alleles present in the genome which were successfully targeted (Eid et al., 2021; Brant et al., 2024). Mutation size and composition can also vary between transgenic events and targeted gene copies/alleles, and multiple tillers or progeny plants must be genotyped to identify chimeric primary transformants (Wang et al., 2018; Eid et al., 2021; Brant et al., 2024). Furthermore, phenotypic outcomes depend on the number and individual expression patterns of the co-mutated alleles, with a high frequency of frameshift mutations often being required to create loss of function phenotypes. Therefore, it is desirable for genotyping methods to not only confirm the presence of mutations but also distinguish between different co-mutation frequencies in the target copies/alleles and detect the size of the created indels (Liang et al., 2018; Eid et al., 2021).

Sugarcane is an interspecific hybrid of *Saccharum officinarum* and *Saccharum spontaneum* and has a highly heterozygous, polyploid genome (2*n* = 100–130), making genotyping of targeted mutations very challenging (Piperidis and D’Hont, 2020). For example, generating a loss of function phenotype of the lignin biosynthesis gene *CAFFEIC ACID O-METHYLTRANSFERASE* (*COMT*) required co-mutagenesis of 107 out of 109 copies (Kannan et al., 2018). To date, only seven studies have shown CRISPR/Cas9 mediated gene editing or transcription modulation in sugarcane, including at the *MG-PROTOPORPHYRIN IX CHELATASE* (*MgCH*), *LIGULELESS 1* (*LG1*), *ACETOLACTATE SYNTHASE* (ALS); *LIM* transcription factor and transgenic *loxP-gusA* loci (Eid et al., 2021; Oz et al., 2021; Zhao et al., 2021; Hooghvorst and Altpeter, 2023; Brant et al., 2024; Laksana et al., 2024; Sosa et al., 2024). In these studies, Sanger and next-generation sequencing (NGS) of target gene PCR amplicons were used to genotype mutant lines. However, for sequencing to provide comprehensive information regarding mutation size, composition, and frequency in edited allelic variants, both a long read length and a high sequencing depth is required, inflating genotyping costs (Stritzler et al., 2022).

As a large numbers of transgenic lines must be screened to identify events with abundant co-editing of the targeted copy/alleles, more affordable genotyping assays are desired. This is demonstrated well with CRISPR-mutagenesis of the *LG1* loci, where a single transgenic sugarcane line out of 78 events exhibited complete co-editing of all 40 *LG1* copies/alleles identified (Brant et al., 2024). In addition, mutation size varied largely between edited lines (Brant et al., 2024). Similarly, Eid et al. (2021) observed 9/53 transgenic sugarcane lines were edited at the *MgCH* loci, only three of which had a sufficient co-editing frequency to induce a chlorophyll depletion phenotype. In both of these studies it was also demonstrated that while free online tools exist for analyzing NGS reads, such as CasAnalyzer (Park et al., 2016), more tailored, in-depth bioinformatic analysis is often required, as many tools are not optimized for use with complex genomes. This highlights the need for reliable, cost-effective methods for mutant screening prior to sequencing.

A number of PCR and electrophoresis-based methods have been used for mutant screening, including cleaved amplified polymorphic sequence (CAPS) assays, T7 endonuclease I assays, digital droplet PCR, high-resolution melt analysis (HRMA), capillary electrophoresis (CE), and annealing at critical temperature PCR (Lomov et al., 2019; Nadakuduti et al., 2019; Thomson et al., 2022). However, many have yet to be optimized for use in highly polyploid species. The only non-sequencing method which has currently been used in combination with CRISPR for mutant screening in sugarcane is CAPS assay (Eid et al., 2021). CAPS assays work on the basis that successful mutagenesis removes a restriction enzyme binding site, allowing non-digested mutant DNA to be visualized by agarose gel electrophoresis (Konovalov et al., 2012). This limits sgRNA selection to those containing a restriction recognition sequence which overlaps the site of Cas-mediated DNA cleavage. Reliability of the assay varies depending on the cleavage efficiency of the restriction enzyme used (Liang et al., 2018). The information content from CAPS assays is also limited, as it is based on the band intensities observed for the digested/undigested products, and does not include precise quantification of co-mutation frequency of the different copies/alleles or indel size (May et al., 2023a). More recently, Cas9 ribonucleoprotein (RNP) assays were developed to overcome the restriction site requirement associated with the CAPS assay. Similar to Cas9 *in vitro* cleavage assays, which confirm sgRNA efficacy, RNP assays involve incubating Cas enzymes and sgRNAs *in vitro* with PCR amplicons of the target region (Bente et al., 2020). As with CAPS assays, uncut products visible following gel electrophoresis suggest the occurrence of targeted edits. While this method has yet to be demonstrated in sugarcane, it has been shown to be effective in wheat (Liang et al., 2018; Gupta and Li, 2021), and could hold potential for sugarcane mutant analysis.

Another method for consideration is CE, which detects indels in PCR amplicons by size fractionation of fluorescently labeled PCR amplicons. Mutation frequencies can then be estimated through quantification of relative fluorescence (Ramlee et al., 2015; Bennett et al., 2020). This method has previously been used for molecular characterization of transgenic sugarcane lines following targeted mutagenesis with transcription activator-like effector nucleases (TALENs) (Jung and Altpeter, 2016). TALEN-mediated mutagenesis typically results in larger insertions or deletions in contrast to CRISPR/Cas9, which frequently results in short indels of 1-3 bp in length (Bortesi et al., 2016; Zhu et al., 2017). While experimental evidence is still lacking for CE in sugarcane CRISPR mutants, it has been successfully demonstrated to detect mutations to a 1 bp resolution in CRISPR-edited tetraploid potato (González et al., 2020). HRMA differs from the forementioned methods as it allows differentiation of amplicon melt temperatures following saturation with double stranded DNA-intercalating dye (Mondini et al., 2011), and does not involve multiple reaction or processing steps. HRMA has demonstrated potential for high throughput screening of targeted edits based on previous reports of its use in wheat and soybean (Mehta et al., 2013; Tan et al., 2016; Camerlengo et al., 2020).

In this study, CE, Cas9 RNP assays, HRMA, and NGS are explored for their potential application in CRISPR/Cas9-mediated mutation screening at six sgRNA target sites located in the *LG1*, *NAC-TRANSCRIPTION FACTOR 108* (*NAC108*)*,* and *TRIGALACTOSYLDIACYLGLYCEROL 5* (*TGD5)* loci in sugarcane. Sugarcane lines selected for the study contained targeted edits in the selected genes which exhibited a range of mutation frequencies (2%–100%) and indel sizes (1–64 bp). The screening methods were deployed in both primary and progeny lines in order to explore their potential for the identification of chimerism. Furthermore, a comparison of the associated costs, benefits, and limitations of each method is included.

## 2 Materials and methods

### 2.1 Target selection and sgRNA design

The *LG1* gene sequence was identified as defined by Brant et al. (2024). In short, the *LG1* sequence was retrieved from the annotated *Sorghum bicolor v3.1.1* reference genome (Accession ABXC03000000) in Phytozome and used to locate the corresponding gene sequence within the sugarcane monoploid genome on CIRAD via tBLASTn (Garsmeur et al., 2018; McCormick et al., 2018). The same procedure was followed to identify the *NAC108* and *TGD5* sequences. Genes were then PCR amplified from sugarcane cultivar CP88-1762 using primers targeted to conserved regions between the sugarcane and sorghum reference genomes. Sequences of cloned PCR amplicons were confirmed with the Sanger method.

**FIGURE 1:**
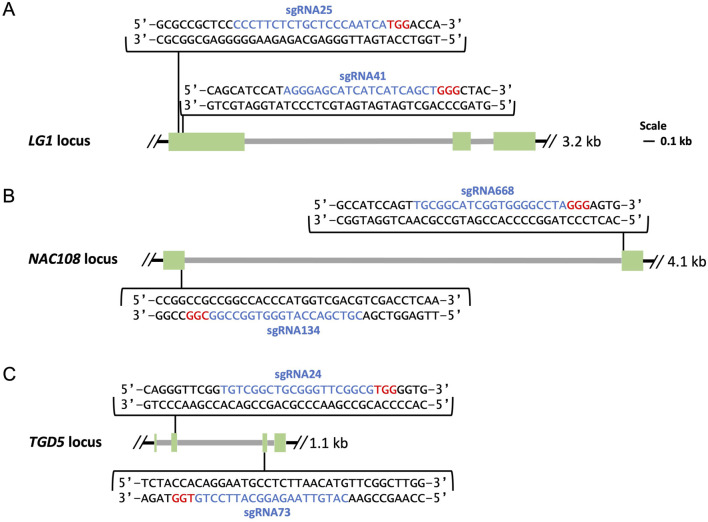
Maps of genomic targets. Exons are shown in green, introns in grey, sgRNA sequences are highlighted in blue followed downstream by 3 bp PAM sequences highlighted in red. **(A)** Location of sgRNA25 and sgRNA41 in exon 1 of the *LG1* loci; **(B)** Location of sgRNA134 and sgRNA668 in exon 1 and exon 2 of the *NAC108* loci; **(C)** Location of sgRNA24 and sgRNA73 in exon 2 and exon 3 of the *TGD5* loci.

SgRNA design was completed using CRISPOR (http://crispor.tefor.net/) and/or Benchling (https://benchling.com), and sgRNAs were selected to target regions which showed conservation between sorghum, the sugarcane monoploid reference, and the target variety. For *LG1,* two sgRNA sites were selected, sgRNA25 and sgRNA41, which were located within 43 bps of each other in exon 1 of the *LG1* loci (Figure 1A) (Brant et al., 2024). For *NAC108*, two sgRNA sites, sgRNA134 and sgRNA668, were selected targeting the first and second exons respectively (Figure 1B). Two sgRNAs were selected for *TGD5,* sgRNA24 and sgRNA73, which targeted the second and third exons, respectively (Figure 1C).

### 2.2 Generation of mutant lines

All mutated lines were created *via* biolistic transfer of the editing reagents and selectable *neomycin phosphotransferase II (nptII*) expression cassette into embryogenic callus as previously described (Brant et al., 2024). To summarize, embryonic callus was induced from leaf whorls cross-sections of sugarcane var. CP88-1762 and bombarded with one of four editing vectors, containing *Cas9*, sgRNA, and *nptII* expression cassettes (Taparia et al., 2012). The minimal cassettes of vectors LG1925 or LG2541 were used to create mutations in *LG1* (Brant et al., 2024). Similarly, minimal cassettes of NA134668 and TG240 were used to induce mutations in *NAC108* and *TGD5*, respectively.

Selection of transgenic lines was completed using culture medium with geneticin and PCR based screening as previously described (Taparia et al., 2012).

### 2.3 Plant material

Plants were maintained in a greenhouse under natural photoperiod conditions with temperature controlled at 25°C–29°C during the day and 20°C–24°C during the night. Drip fertigation was used to irrigate and fertilize all pots with Miracle Gro All Purpose Plant Food (ScottsMiracle-Gro, OH, United States).

### 2.4 Primer design

Where possible, primer pairs which previously showed success for target gene amplification in sugarcane var. CP88-1762 were retrieved from the literature and used within this study (Supplementary Table S1) (Brant et al., 2024).

For all other primers, the following considerations were made. Conserved primer annealing locations within exons where prioritized to maximize co-amplification of different copies/alleles and minimize size variation of wild type (WT) PCR amplicons. For the *LG1* gene, forty copies and their respective single nucleotide polymorphisms (SNPs) have previously been reported in sugarcane var. CP88-1762 (Brant et al., 2024). Therefore, all *LG1* primers were designed to fall within conserved regions. As detailed copy information was not available for the other genes targeted, a BLAST search of each target gene sequence was performed against the *S*. *officinarum* cultivar LA purple genome (ASM2063173v1; NCBI). Any hits showing more than 75% query cover were aligned and primers were designed to anneal to conserved regions, unless otherwise stated. To minimize unspecific amplification, all primer pairs were confirmed against sugarcane’s closest diploid relative, *S. bicolor* (taxid:4,558), using the PrimerBLAST tool (https://www.ncbi.nlm.nih.gov/tools/primer-blast/). Selected primers were subsequently ordered as standard oligonucleotides from Eurofins Genomics (KY, United States).

### 2.5 DNA extraction and PCR

Genomic DNA was extracted following the cetyltrimethylammonium bromide (CTAB) method, as previously described, with minor modifications (Murray and Thompson, 1980).

PCR reactions were completed using Q5^®^ High-Fidelity DNA Polymerase (NEB, MA, United States) following the manufacturers guidelines for reaction set up without high G/C enhancer.

### 2.6 Next-generation sequencing (NGS)

#### 2.6.1 NGS targets and sample preparation

For *tgd5* lines, two regions covering the *TGD5* exon 2 sgRNA24 target site (227 bp amplicon) and *TGD5* exon 3 sgRNA73 target site (228 bp amplicon) were amplified. Primer pairs P1/P2 and P3/P4 (Supplementary Table S1) were used, respectively. Reaction conditions were as listed for PCR1 in Supplementary Table S2.

For *nac108* lines, a 230 bp amplicon containing the *NAC108* exon 1 sgRNA134 target site was amplified using primer pair P5/P6 (Supplementary Table S1), with PCR1 reaction conditions (Supplementary Table S2), and a 210 bp amplicon containing the *NAC108* exon 2 sgRNA668 target site was amplified using primer pair P7/P8 (Supplementary Table S1) with PCR2 reaction conditions (Supplementary Table S2).

For lg*1* lines, a 246 bp region of *LG1* exon 1 was amplified which covered both sgRNA target sites (sgRNA25 and sgRNA41) as previously described by Brant et al. (2024) using primer pair P9/P10 (Supplementary Table S1), following reaction conditions listed for PCR3 (Supplementary Table S2).

A GeneJET PCR Purification Kit (K0701; Thermo Fisher Scientific) was using to complete PCR purification of all reactions. Samples were subject to CRISPR Amplicon NGS using Illumina MiSeq at the CCIB DNA Core Facility, Massachusetts General Hospital (MA, United States). During library preparation, NGS adaptors and unique barcodes were added to amplicons at the 5′ and 3′ ends, generating paired end reads.

#### 2.6.2 NGS analysis for indel size

For *TGD5* sgRNA24, NGS fastq files were analyzed to identify the 5′primer (P1) in the forward (F) reads. If an exact match was found, the reads were then searched for a conserved sequence (CS1; Supplementary Table S1) located 120 bp downstream of P1 in the WT reads. Any reads which contained both the primer and conserved sequence and deviated from the expected length for WT reads were recorded to contain an indel of the deviation size. Deviations which appeared at less than a 1% frequency (of total reads containing both CS1 and P1) were not considered based on the level of background noise observed in the WT sample, most likely representing sequencing error. This analysis method is forthwith referred to as conserved sequence (CS) analysis.

Similarly, for *TGD5* sgRNA73, fastq files were analyzed using P3 and CS2 (105 bp), where *NAC108* sgRNA134 and sgRNA668 were analyzed using P5/CS3 (130 bp) and P8/CS4 (110 bp), respectively (Supplementary Table S1). For *LG1*, as both sgRNA sites considered were contained within the same target amplicon, they were analyzed together using P9 and CS5 (128 bp; Supplementary Table S1) as previously described by (Brant et al., 2024). The code used for CS analysis is included in Supplementary File 1.

#### 2.6.3 NGS analysis for indel frequency with Cas-Analyzer

As the CS analysis was unable to analyze indel frequency at individual sgRNA sites in the lg*1* lines or highlight sequence information, fastq files were also analyzed using the free online Cas-Analyzer (CA) tool (Park et al., 2016). CA searches for two conserved 12 bp indicator sequences within reads. If both are found, the sgRNA target site is analyzed for the presence of indels. The distance between the indicator sequences and target site is defined per target, allowing smaller regions to be analyzed than with CS. The parameters used for CA of each target are listed in Supplementary Table S3.

Read count was extracted for each indel type to calculate frequency of each indel size. Reads that had a frequency of less than 0.015% were not included in the calculation, based on the level of background noise observed in the WT sample, most likely representing sequencing error. For *LG1*, CS analysis indicated a large deletion in some lines. However, these large deletions were not detected in the initial Cas Analyzer (CA) output for *LG1*. Therefore, CA was rerun for sgRNA41 using an R of 70 bp as defined by Brant et al. (2024). These reads were then added to the total reads for both sgRNA25 and sgRNA41 when calculating indel frequencies.

### 2.7 Fragment size analysis through capillary electrophoresis

#### 2.7.1 Sample preparation

Amplicons used in fragment size analysis were generated using the same primers and conditions listed in section 2.4. for NGS, with the exception that the forward primer for each reaction contained a 5′ 6-FAM label (listed as primers P11-P15, Supplementary Table S1), and light exposure of samples was avoided to the extent possible.

Following amplification, each PCR product was diluted to a concentration of 30 ng/μL using Nuclease-Free H_2_O. Samples were prepared with the following: 10 μL Hi-Di™ Formamide (4,401,457; Applied Biosciences, CA, United States), 0.5 μL GeneScan™ 600 LIZ™ dye Size Standard v2.0 (4,408,399; Applied Biosciences), and 2.0 μL diluted PCR product.

#### 2.7.2 Capillary electrophoresis

CE was completed on an ABI 3730xl DNA analyzer (Applied Biosystems) using Eurofins Genomics Ready2Load Fragment Analysis Service. Standard run parameters were used, as defined by Eurofins Genomics.

Amplicon peak size and height were determined using the Peak Scanner Software v2.0 (Thermo Fisher Scientific). Estimated co-editing percentage was calculated as the (sum of all amplicon peak heights–wild type peak height)/(sum of all amplicon peak heights)*100. Where multiple peaks were observed in the WT sample, all peaks of those sizes were considered WT in mutated samples.

### 2.8 Cas9 RNP cleavage assay

#### 2.8.1 Template DNA amplification

A 1048 bp region of the *TGD5* gene was amplified (Q5^®^ High-Fidelity DNA Polymerase; NEB) from respective *tgd5* lines using primer pair P16/P17 (Supplementary Table S1), following reaction conditions PCR5 (Supplementary Table S2). A 955 bp region of the *LG1* gene was amplified from lg*1* lines using primer pair P18/P19 for investigation of the sgRNA25 site, and a 647 bp region was amplified using primer pair P9/P19 for the sgRNA41 site (Supplementary Table S1) both using reaction conditions PCR3 (Supplementary Table S2). For investigation of *nac108* lines, two regions were amplified. The exon 1 sgRNA134 target site was included in a 647 bp amplicon using primer pair P20/P21 (Supplementary Table S1), following reaction conditions PCR6 (Supplementary Table S2). The exon 2 sgRNA668 target site was included in a 614 bp amplicon using primer pair P22/P23 (Supplementary Table S1), following reaction conditions PCR7 (Supplementary Table S1).

#### 2.8.2 sgRNA synthesis

To enable amplification of the sgRNA scaffold using overlapping PCR (Q5^®^ High-Fidelity DNA Polymerase; NEB), a forward primer was designed for each sgRNA. These contained a T7 promoter sequence followed by the desired sgRNA sequence as a 5′ overhang, along with appropriate buffer regions, and a 20 bp sequence homologous to the scaffold. Primers P24 and P25 were designed to amplify *TGD5* sgRNA24 and sgRNA73 with scaffold, respectively. Primers P26 and P27 were designed to amplify *NAC108* sgRNA134 and sgRNA668 with scaffold, respectively (Supplementary Table S1). For *LG1*, primers P28 and P29 were each designed to amplify sgRNA25 and sgRNA41 with scaffold, respectively (Supplementary Table S1). A single reverse primer, P30 (Supplementary Table S1), was utilized for all reactions. Six reactions were undertaken in total, one for each target analyzed, all following the conditions listed as PCR4 in Supplementary Table S2.

PCR products were size fractionated by electrophoresis on a 1% agarose gel. The desired product for each reaction (125 bp) was extracted and purified using a GeneJET Gel Extraction Kit (K0692; Thermo Fisher Scientific). A HiScribe™ T7 Quick High Yield RNA Synthesis Kit (E2050S; NEB) was used to complete *In vitro* transcription and DNase I treatment of each sgRNA/scaffold combination. RNA cleanup was completed using phenol/chloroform followed by ethanol precipitation, with minor modification (Toni et al., 2018).

#### 2.8.3 RNP cleavage assay

For *LG1* and *TGD5*, the *in vitro* cleavage assays were performed using 100 ng of template DNA, 100 ng sgRNA/scaffold, 200 ng Cas9 protein (PNA Bio, CA, United States), and 1 μL NEB buffer 3.1 in a 10 μL reaction. For *NAC108*, 300 ng Cas9 protein was used. Reactions were incubated at 37°C for 16 h, followed by heat inactivation at 65 °C for 10 min, prior to electrophoresis on a 1% agarose gel.

#### 2.8.4 Scoring of RNP assay

Mutation scoring was completed based on comparison between cleaved and non-cleaved PCR amplicons. ImageJ (Rasband, 2018) was used to measure the intensity (mean grey value; *GV*) of each band present on the gel. When a single cleaved product was present, the difference between the WT and mutant products were calculated as (Δ*GV* = mean *GV* cleaved product–mean *GV* non-cleaved product). A difference of < - 90 was given a score of 0 and taken to represent no mutations. When both a cleaved and non-cleaved product were present, but the signal representing the cleaved amplicon appeared brighter (Δ*GV* = - 90 to - 31), this was scored 1 and considered to be co-mutated at < 40% frequency. When both cleaved and non-cleaved products appeared at equal strengths (Δ*GV* = −30 to 30), a score of 2 was given and considered to be ∼50% co-mutated. If the non-cleaved product was brighter or only a non-cleaved product was present, these lines would be scored 3 (Δ*GV* = 31 to 90; >60% mutated) or 4 (Δ*GV* = >90; 100% mutated), respectively. A score of 5 was given to lines which exhibited no un-cleaved product or a second product which did not match the expected size of a cleaved amplicon.

Where two cleaved products were present, an average of the mean *GV* was calculated for cleaved products and this was used in the Δ*GV* calculation. As this decreased the intensity of the Δ*GV* for the cleaved products, Δ*GV* score ranges were adjusted to 0 = <-60, 1 = -60 to – 21, 2 = −20 to 20, 3 = 21 to 60, 4 = >60.

### 2.9 Melt curve and high-resolution melt (HRM) analyses

#### 2.9.1 HRM primer design and melt curve analysis

Where possible, primers were designed to amplify regions of 70–110 bp to enable optimal discrimination between mutant and WT samples (Cousins et al., 2013). Two primer pairs were initially tested per target (Supplementary Table S4). The best primer pair was selected based on performance in a standard melt curve reaction using 10.0 μL SsoAdvanced Universal SYBR Green Supermix (BioRad, CA, United States), 0.5 μM F Primer, 0.5 μM R Primer, and 100 ng of WT DNA (20.0 μL total volume reaction). Run conditions were the same for all targets/primer pairs, as defined for Melt Curve in Supplementary Table S2. If neither primer pair performed well, additional pairs were designed and subject to the same screening (Supplementary Table S4). Mutant samples were subsequently run in triplicate on a Rotor-Gene Q instrument (Qiagen) and visualized using the Melt Curve Module on the Q-Rex Software (Qiagen).

#### 2.9.2 HRM analysis

Type-it HRM PCR Kit (Cat #206544; Qiagen) was used for all HRM reactions, following manufacturers recommendations with minor modifications. A 25.0 μL total volume reaction was used, containing 12.5 μL 2x HRM PCR Master Mix, 0.7 μM F Primer, 0.7 μM R Primer, and 100 ng of DNA. Each sample was run in triplicate on a Rotor-Gene Q instrument (Qiagen). The presence of primer-dimers and/or non-specific amplification was assessed using melt curve data, visualized with the Basic Module on the Q-Rex Software (Qiagen). Run conditions were as defined for HRMA in Supplementary Table S2. Following the initial run for each target, melt temperature range was decreased where possible to decrease run times. Data was visualized using the HRM Analysis Module on the Q-Rex Software (Qiagen). HRMA melting data was normalized by adjusting beginning and end fluorescence signals to the same level for all samples at a given target (Supplementary Table S5). Two WT samples were included in each run, in triplicate, and defined as a “WT genotype” used for baseline comparison when generating difference curves (fluorescence difference vs temperature).

### 2.10 Price comparison

Prices were retrieved from online catalogs, shown in Supplementary Table S6. Where components were available in bulk, the smallest size that would allow processing of 96 samples was selected. Total component cost was divided by the number of reactions that could be completed with that component to calculate component cost per sample. For each analysis method, component costs per sample were added together to calculate total cost per sample. Cost of shipping, pipette tips, equipment running, maintenance, labor, and optimizations were not included due to variability between locations and experiments.

## 3 Results

### 3.1 Selection of mutant lines for comparison of genotyping methods

For analysis of *LG1* sgRNA target sites, progeny of seven lg*1* mutant lines (L13, L7, L11, L17, L26, L35, and L44), previously shown to contain a range of edit frequencies at each *LG1* target site, were selected for analysis (Brant et al., 2024). Three progeny plants per edited line were selected, known as L13a-c, L7a-c, L11a-c, L17a-c, L26a-c, L35a-c, and L44a-c.

For *NAC108* sgRNA target sites, progeny of six *nac108* mutant lines (*nac-*3, *nac-*4, *nac-*6, *nac-*8, *nac*-9, and *nac-*11) containing a range of edit frequencies at each sgRNA target site were generated. One progeny plant was selected per edited line, known as N3a, N4a, N6a, N8a, and N11a. Twelve *tgd5* mutant lines were generated (T32, T34, T41, T47, T53, T55, T77, T81, T82, T205, T206, and T208) and primary lines were used for analysis.

### 3.2 Confirmation of mutation frequency by NGS

#### 3.2.1 NGS mutation frequencies observed in *nac108* lines

The number of reads analyzed in each NGS analysis for *nac108* lines are listed in Supplementary Table S7. At the *NAC108* sgRNA134 site, the frequency of non-WT reads ranged between 29.4% and 100.0% between lines. Within this the largest indel was a 28 bp deletion in ∼11.6% of the reads of N8a (Table 1). Indel size predicted by CA matched the CS analysis in all cases for this target. However, the frequency ranged more dramatically between CS and CA than at any other target analyzed, with as high as 10.6% variation observed (N11a, ‘A’ insert; Table 1) between the two analyses for the same indel. This difference was likely due to the distinctive parameters used causing variation in conserved sequence vs indicator sequence recognition, leading to different reads being highlighted in each analysis.

**TABLE 1:** Comparison of genotyping analysis outcomes for *NAC108* sgRNA134 and sgRNA668 assays, including mean melt peak temperatures (MC; melt curve), the maximum fluorescence difference from WT extracted from HRMA difference plots, and Cas9 RNP assay scores. Alongside this, the frequency of non-WT peaks or reads is shown for capillary electrophoresis (CE), NGS Cas Analyzer (CA) and conserved sequence analysis (CS) for size and frequency of individual indels.

Target	Line id	MC mean peak temp/°C	HRM max. Fluor. Diff. to WT	Cas9 RNP score	Freq. Non-WT reads or peaks			Breakdown of indels						
								CS NGS		CA NGS		CE		Indel sequence extracted from CA
					CS NGS	CA NGS	CE	Size diff. To WT/bp	Freq./%	Size diff. To WT/bp	Freq./%	Size diff. To WT/bp	Freq./%	
*NAC108* *sgRNA 134*	WT	92.0	1	0	0.0	0.0	0.0	-	-	-	-	-	-	*-*
	N3a	91.9	7	1	30.6	29.4	22.3	+1	29.9	+1	25.9	+1	22.3	*a or t*
	N4a	91.8	8	2	37.0	34.8	31.5	+1	34.3	+1	27.7	+1	31.5	*a, c, or t*
	N6a	91.8	8	2	41.5	40.0	37.6	+1	41.3	+1	33.8	+1	37.6	*a or t*
	N8a	91.5	20	4	100.0	100.0	100.0	+1	64.2	+1	56.6	+1	51.4	*a or t*
								+2	7.3	+2	5.3	+2	11.0	*aa*
								−2	9.0	−2	8.2	−2	11.9	*gg*
								−10	7.9	−10	7.3	−10	10.6	*ggccacccat*
								−28	11.6	−28	11.5	−28	15.2	*cgg​cgg​caa​ggt​cgg​ctt​ctc​cgg​ccg​c*
	N9a	91.9	7	2	35.7	34.8	30.2	+1	34.8	+1	30.8	+1	30.2	*a or t*
	N11a	91.8	20	4	100.0	100.0	100.0	+1	67.6	+1	57.0	+1	70.2	*a*
								−1	7.9	−1	6.9	−1	7.5	*g*
								−3	8.0	−3	8.9	−2	7.0	*cgg*
								−7	7.9	−7	7.4	−6	7.9	*ccggcca*
								−14	8.6	−14	7.1	−13	7.4	*gccggccacccatg*
*NAC108* *sgRNA 668*	WT	85.8	1	0	0.1	0.1	0.0	-	-	-	-	-	-	*-*
	N3a	85.8	8	1	12.7	12.4	10.9	+1	11.1	+1	10.3	+1	10.9	*a or t*
	N4a	85.8	4	0	8.9	8.6	9.3	+1	6.6	+1	6.2	+1	9.3	*a, g, or t*
								−1	1.1	−1	1.0	−1	-	*g*
	N6a	85.8	2	0	0.2	0.2	0.0	-	-	-	-	-	-	*-*
	N8a	80.9, 85.3	19	3	49.9	49.6	49.8	+1	17.0	+1	16.5	+1	16.7	*a or t*
								−1	2.0	−1	1.8	−1	4.1	*g*
								−2	15.1	−2	14.4	−2	14.5	*gg*
								−12	13.8	−12	13.4	−12	14.5	*ccactccctagg*
	N9a	85.8	1	1	1.7	1.5	0.0	-	-	-	-	-	-	*-*
	N11a	85.0	17	2	45.2	44.9	43.4	+1	25.5	+1	24.7	+1	25.2	*a or t*
								−1	3.0	−1	2.8	−1	4.4	*c or g*
								−7	14.4	−7	13.7	−7	13.8	*gccccac*

Temp., temperature; Fluor., fluorescence; Diff., difference; Freq., frequency; RNP, Scoring – 0 = no mutations, 1 = <40% mutated, 2 = ∼50% mutated, 3 = >60% mutated, 4 = 100% mutated, 5 = 100% mutated with large indels.

At *NAC108* sgRNA668, the frequency of non-WT reads for the different lines ranged between 8.6% and 49.9% (Table 1). Here, the indel sizes predicted by CA also matched the CS analysis. However, the largest indel was a 12 bp deletion in ∼13.6% reads in line N8a. Unlike at the sgRNA134 site, frequencies only varied by 1.5% between the two analyses for each indel (Table 1).

#### 3.2.2 NGS mutation frequencies observed in lg*1* lines

The number of reads analyzed in each NGS analysis for lg*1* lines were listed in Supplementary Table S8. A range of mutation frequencies were observed in the lg*1* lines with the CS analysis, with frequencies of non-WT reads ranging from 9.6% to 95.6% between lines (Table 2; Supplementary Table S9). As the CS analysis considered both *LG1* sgRNA target sites together, where CA analyzed them separately, overall frequency of non-WT reads could not be compared between the two analyses for this target. However, CA provided an indication of mutation frequency at each sgRNA site individually, giving a breakdown of indel size at each site. While the majority of size differences observed with the CS analysis matched indel sizes observed with CA, some discrepancies were observed. For example, in L17a and L17c a size deviation of – 7 bp was observed with the CS analysis, where no 7 bp indels were observed at either sgRNA site with CA. This suggests some reads contained a combination of indels at both the sgRNA41 and sgRNA25 sites simultaneously. This was also observed in L44a-c and L11a and L11b (Table 2; Supplementary Table S9).

**TABLE 2:** Comparison of genotyping analysis outcomes for *LG1*, including mean melt peak temperatures (MC; melt curve), the maximum fluorescence difference from WT extracted from HRMA difference plots, capillary electrophoresis (CE) data, and conserved sequence (CS) NGS analysis for indel length of fragments containing both sgRNA sites together. Cas9 RNP assay scores and Cas Analyzer (CA) NGS data are provided for each sgRNA site individually.

	MC	HRMA	CE			CS NGS				Cas9 RNP	CA NGS			
Line id	Mean peak temp/°C	Max fluor. Diff. to WT	Overall indel freq./%	Indel size/bp	Indel freq./%	Freq. Non-WT reads/%	Size diff. To WT/bp	Freq./%	Target sgRNA	Score	Freq. Non-WT reads/%	Size diff. To WT/bp	Freq./%	Indel sequence
WT	89.1	2	0.0	-	-	0.1	-	-	25	0	0.0	-	-	-
									41	0	0.0	-	-	-
L7a	89.3	4	43.7	+1	32.4	43.0	+1	35.8	25	1	0.0	-	-	-
				−42	11.3		−42	7.2	41	2	43.1	+1	34.6	*a or t*
												−42	6.9	*tcatggagcaggagagca+*
L11a	89.0	15	84.0	+1	20.0	78.6	+1	29.0	25	2	33.2	+16	13.9	*tcc​ct--a>c---gca​cca​cca​c--a*
				−1	13.3		−1	13.6				−12	18.4	*aaccctcgtctc*
				−11	14.6		−11	18.3	41	4	78.8	+1	45.4	*a or t*
				−12	14.5		−54	17.7				−1	13.4	*g*
				−54	21.5							−16	17.2	*atcgggtcgactacta*
L13a	89.0	4	18.1	+1	18.1	17.5	+1	17.5	25	1	17.7	+1	17.3	*a*
									41	0	0.1	-	-	-
L17a	89.0	11	63.0	+2	13.3	74.2	+2	15.9	25	2	36.5	+1	16.3	*a*
				−1	7.5		+1	8.7				−1	9.0	*a*
				−2	5.3		−1	8.7				−6	7.8	*actaac*
				−6	8.4		−2	7.8	41	3	56.6	+1	10.2	*a or t*
				−7	10.2		−6	8.3				−1	10.1	*g*
				−8	8.7		−7	9.1				−2	9.0	*ac*
				−42	9.7		−8	7.9				−6	10.7	*gtcgac*
							−42	7.6				−8	9.0	*cgactact*
												−42*	8.7	*tca​tgg​agc​agg​aga​gca​g*+
L26a	89.0	8	55.1	+1	20.7	49.4	+1	16.7	25	1	18.1	−3	9.1	*cta*
				−3	8.2		−3	7.8				−4	8.3	*taac*
				−5	8.0		−4	8.4	41	2	33.0	+1	16.0	*a or t*
				−14	8.3		−14	7.9				−14	7.6	*gggtcgactactac*
				−23	9.9		−23	8.3				−23	8.1	*cat​cgg​gtc​gac​tac​tac​ta+*
L35a	89.2	2	10.9	−8	10.9	11.4	−8	11.3	25	0	0.0	-	-	*-*
									41	1	11.3	−8	10.9	*tcgacta - - - c > t - a*
L44a	89.5	16	100.0	−22	10.9	93.1	−22	14.4	25	3	88.7	−21	12.0	*cct​tct​ctg​ctc​cca​atc​atg*
				−42	23.0		−42	21.2	41	4	93.1	−1	14.8	*a*
				−43	54.9		−43	47.9				−42*	20.1	*tca​tgg​agc​agg​aga​gca​g+*
				−46	11.2		−46	9.4				−43*	46.5	*atc​atg​gag​cag​gag​agc​a+*
												−46*	9.1	*tca​tgg​agc​agg​aga​gca​g+*

Temp., temperature; Fluor., fluorescence; Diff., difference; Freq., frequency; *, deletion spanned across both sgRNA, sites; RNP, Scoring – 0 = no mutations, 1 = <40% mutated, 2 = ∼50% mutated, 3 = >60% mutated, 4 = 100% mutated, 5 = 100% mutated with large indels.

In addition, for lines L11a and L11b, a 16 bp insertion and 16 bp deletion was observed at the sgRNA25 and sgRNA41 sites, respectively, which did not match any size deviations observed in the CS analysis, highlighting potential miscalling. With CA, relatively short indicator ranges were used for the *LG1* sgRNA targets (35 bp; Supplementary Table S3). While a larger *R* (70 bp) allowed indels between 40 and 50 bp to be identified, it greatly reduced the total number of reads that were included in the analysis and led to indels <40 bp not being identified. Overall, the largest indel observed was in L11a and L11b, where a 54 bp deviation from the WT read was observed in the CS analysis in ∼20.0% of the read, representing a 54 bp deletion. However, this indel was not detectable using CA (Table 2; Supplementary Table S9).

#### 3.2.3 NGS mutation frequencies observed in *tgd5* lines

The number of reads analyzed in each NGS analysis for *tgd5* lines were listed in Supplementary Table S7. At the *TGD5* sgRNA73 site, the frequency of non-WT reads ranged between 2.7% and 100%, and the CS and CA analyses matched very closely, with all lines except T53 showing the same size deviations in the CS as indel size with CA. Within T53, the difference between the two analyses was observed as CS called a 22 bp deviation in 11.5% of reads, where CA predicated a 21 bp deletion in 11.2%. In addition, the frequency of indels observed also matched between the two analyses, with <2% difference between analysis of each indel (Table 3). Out of all lines and targets analyzed, line T82 showed the lowest mutation frequency editing at sgRNA73, with an insertion of 2 bp appearing in only ∼2.7% of reads.

**TABLE 3:** Comparison of genotyping analysis outcomes for *TGD5* sgRNA73 assays, including mean melt peak temperatures (MC; melt curve), the maximum fluorescence difference from WT extracted from HRMA difference plots, and Cas9 RNP assay scores. Alongside this, the frequency of non-WT peaks or reads is shown for capillary electrophoresis (CE), NGS Cas Analyzer (CA) and conserved sequence analysis (CS) for size and frequency of individual indels.

Line id	MC mean peak temp/°C	HRM max fluor. Diff. to WT	Cas9 RNP score	Freq. of Non-WT reads or peaks/%			Breakdown of indels						
							CS NGS		CA NGS		CE		Indel sequence extracted from CA
				CS NGS	CA NGS	CE	Size diff. To WT/bp	Freq./%	Size diff. to WT/bp	Freq./%	Size diff. to WT/bp	Freq./%	
WT	82.3	1	0	0.1	0.0	0.0	-	-	-	-	-	-	*-*
T32	80.0, 82.2	28	4	100.0	99.9	77.7	+1	36.5	+1	34.9	+1	35.8	*g or t*
							+3	24.3	+3	23.1	+3	20.6	*atg*
							−1	16.7	−1	16.0	-	-	*g*
							−3	22.3	−3	21.5	−3	21.3	*aat*
T34	81.8	21	5	99.9	99.9	89.2	+1	49.6	+1	47.8	+1	42.4	*g or t*
							+2	33.1	+2	31.8	+2	29.5	*gg*
							+3	8.8	+3	8.3	+3	6.6	*ggg*
							−18	8.4	−18	8.1	−18	10.7	*cacaggaatgcctcttaa*
T41	80.2, 81.7	30	3	57.5	56.9	36.5	+1	13.5	+1	12.2	+1	12.5	*g or t*
							−1	19.3	−1	18.0	-	-	*c or g*
							−5	9.6	−5	9.0	−5	10.3	*ggaat*
							−18	13.9	−18	13.3	−18	13.8	*taccacaggaatgcctct*
T47	82.0	11	1	37.6	37.6	32.3	+1	26.3	+1	24.4	+1	20.3	*g*
							−36	10.6	−36	10.5	−36	11.8	*gct​ttt​cta​cca​cag​gaa​tg+*
T53	77.2, 81.8	20	2	53.6	53.6	47.0	+1	11.2	+1	10.6	+1	8.7	*g*
							+2	7.2	+2	6.8	-	-	*gg*
							−1	4.9	−1	4.6	-	-	*g*
							−18	18.7	−18	18.3	−18	19.0	*cacaggaatgcctcttaa*
							−22	11.5	−21	11.2	−22	12.7	*ctt​ttc​tac​cac​agg​aat​gcc​t*
T55	82.3	-	0	0.1	0.0	0.0	-	-	-	-	-	-	*-*
T77	82.0	4	1	16.4	16.1	14.7	+1	9.1	+1	8.6	+1	7.9	*g*
							−2	7.3	−2	6.9	−2	6.8	*ag*
T81	82.0	12	2	44.5	44.3	37.4	+1	31.7	+1	30.3	+1	27.3	*a, g. or t*
							−11	12.8	−11	12.4	−11	10.1	*caggaatgcct*
T82	82.1	-	1	2.9	2.7	0.0	+2	2.8	+2	2.6	-	-	*tt*
T205	82.0	11	3	71.7	71.7	63.6	+1	59.1	+1	57.2	+1	50.4	*g*
							+4	12.6	+4	11.9	+4	13.2	*tgtt*
T206	82.0	9	2	43.8	43.4	39.8	+1	28.7	+1	26.8	+1	23.1	*a or g*
							−6	14.9	−6	14.0	−6	16.6	*acagga*
T208	82.2	-	0	0.1	0.1	0.0	-	-	-	-	-	-	*-*

Temp., temperature; Fluor., fluorescence; Diff., difference; Freq., frequency; RNP, Scoring – 0 = no mutations, 1 = <40% mutated, 2 = ∼50% mutated, 3 = >60% mutated, 4 = 100% mutated, 5 = 100% mutated with large indels.

In comparison, at the *TGD5* sgRNA24 site the frequency of non-WT reads ranged from 10.9% to 100.0% (Table 3; Supplementary Table S10). The largest size deviation observed in the CS analysis was a 63 bp dropout in 12.9% reads for line T34, which was also predicted as a 64 bp deletion in 12.8% reads by CA. The frequency of different size indels were predicted within 3% of each other by both CS and CA (Table 4). Line 205 showed the largest deviation in indel size between CS and CA observed out of any target site, with a 24 bp size deviation observed with CS in 27.9% of the reads and a 21 bp deletion in 27.3% of the reads with CA (Table 4).

**TABLE 4:** Comparison of genotyping analysis outcomes for *TGD5* sgRNA24 assays, including mean melt peak temperatures (MC; melt curve), the maximum fluorescence difference from WT extracted from HRMA difference plots, and Cas9 RNP assay scores. Alongside this, the frequency of non-WT peaks or reads is shown for capillary electrophoresis (CE), NGS Cas Analyzer (CA) and conserved sequence analysis (CS) for size and frequency of individual indels.

Line id	MC mean peak temp./°C	HRM max fluor. Diff. to WT	Cas9 RNP score	Freq. of Non-WT reads or peaks/%			Breakdown of indels						
							CS NGS		CA NGS		CE		Indel sequence extracted from CA
				CS NGS	CA NGS	CE	Size diff. to WT/bp	Freq./%	Size diff. to WT/bp	Freq./%	Size diff. to WT/bp	Freq./%	
WT	89.1	2	0	0.1	0.0	0.0	-	-	-	-	-	-	-
T32	82.8, 87.7	58	4	96.2	94.1	91.9	+1	40.3	+1	40.1	+1	38.9	*a, g, or t*
							−9	11.3	−9	10.9	−9	10.0	*gcgggttcg*
							−12	11.0	−12	10.5	−12	12.0	*gggttcggcgtg*
							−17	21.4	−17	26.4	−18	16.6	*gggttcggcgtggggtg or ttcggcgtggggtgggg*
							−18	5.9			−19	8.1	
							−35	6.3	−35	6.2	−36	6.3	*ggg​ttc​ggt​gtc​ggc​tgc​ggg​ttc​ggc+*
T34	88.1	60	5	99.8	99.9	100.0	+1	49.4	+1	46.9	+1	48.8	*a, c, or t*
							+2	15.3	+2	14.6	+2	15.7	*tt*
							−17	22.1	−17	21.5	−18	18.0	*gggttcggcgtggggtg*
							−63	12.9	−64	12.8	−64	17.4	*-*
T41	83.9, 88.1	60	4	97.0	96.7	93.0	+1	44.6	+1	47.7	+1	45.9	*a or t*
							+3	4.8	+3	5.0	+3	5.2	*gct*
							-	-	-	-	+47	6.4	*-*
							−9	18.9	−9	20.7	−9	21.8	*gcgggttcg*
							−17	14.4	−17	16.2	−18	13.8	*gggttcggcgtggggtg*
T47	88.6	60	4	94.6	94.8	88.7	+1	64.5	+1	61.9	+1	61.3	*a or t*
							+2	9.3	+2	8.7	+2	9.5	*gg*
							−34	20.5	−34	20.2	−31	6.6	*gtc​ggc​tgc​ggg​ttc​ggc​gtg​ggg​tg+*
											−35	11.2	
T55	89.2	58	1	10.9	10.7	10.0	+2	10.8	+2	10.2	+2	10.0	*tt*
T77	89.0	60	1	45.3	45.0	36.0	+1	15.0	+1	14.1	+1	14.3	*c or t*
							−3	11.3	−3	10.8	−3	10.8	*gtt*
							−18	18.9	−18	18.2	−19	10.9	*gggttcggcgtggggtgg*
T81	89.0	60	1	13.1	12.8	12.8	+1	4.5	+1	4.2	+1	5.4	*a*
							+2	8.5	+2	8.0	+2	7.4	*aa*
T82	89.0	60	1	18.2	17.9	10.8	+1	9.7	+1	9.0	+1	10.8	*a or t*
							−1	8.5	−1	7.9	-	-	*g*
T205	86.2, 89.0	60	4	90.0	90.2	82.6	+1	43.4	+1	41.6	+1	17.1	*a or t*
							+3	18.6	+3	17.8	+3	45.3	*ccc*
							−24	27.9	−21	27.3	−21	20.2	*cgg​gtt​cgg​cgt​ggg​gtg​ggg​ttt*
T208	83.3, 88.8	60	2	50.9	50.6	37.7	+1	26.2	+1	24.1	+1	24.9	*a or t*
							−1	10.7	−1	10.0	-	-	*g*
							−10	12.5	−10	12.1	−10	12.8	*ctgcgggttc*

Temp., temperature; Fluor., fluorescence; Diff., difference; Freq., frequency; RNP, Scoring – 0 = no mutations, 1 = <40% mutated, 2 = ∼50% mutated, 3 = >60% mutated, 4 = 100% mutated, 5 = 100% mutated with large indels.

### 3.3 Fragment size analysis

#### 3.3.1 CE indel estimation accuracy with a single WT peak (*NAC108* and *LG1)*

**FIGURE 2:**
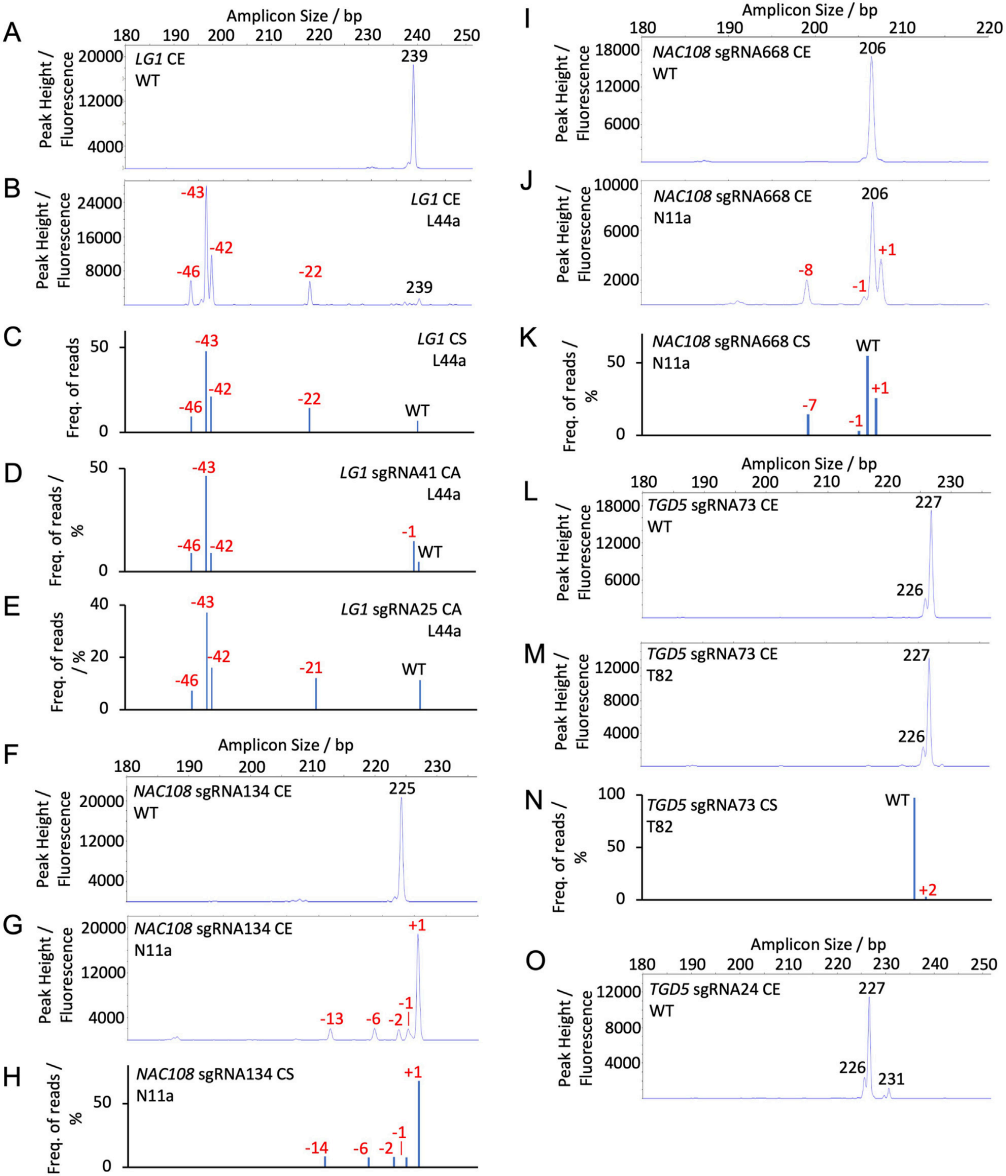
Capillary electrophoresis (CE) peak fluorescence graphs and NGS (conserved sequence, CS, and Cas Analyzer, CA) read frequency graphs for WT and select mutant samples. The size of WT peaks/amplicons is labeled by bp in black. Indels are labeled by size in red **(A)** CE *LG1* sgRNA25 and sgRNA41 WT; **(B)** CE *LG1* assay for line L44a; **(C)** CS *LG1* NGS for line L44a; **(D)** CA *LG1* sgRNA41 NGS for line L44a; **(E)** CA *LG1* sgRNA25 NGS for line L44a; **(F)** CE *NAC108* sgRNA134 WT; **(G)** CE *NAC108* sgRNA134 assay for line N11a; **(H)** CS *NAC108* sgRNA134 NGS for line N11a; **(I)** CE *NAC108* sgRNA668 WT; **(J)** CE *NAC108* sgRNA668 assay for line N11a; **(K)** CS *NAC108* sgRNA668 NGS for line N11a; **(L)** CE *TGD5* sgRNA73 WT; **(M)** CE *TGD5* sgRNA73 assay for line T82; **(N)** CS *TGD5* sgRNA73 NGS for line T82; **(O)** CE *TGD5* sgRNA24 WT.

For *NAC108* sgRNA134, the 230 bp WT amplicon resulted in a single 225 bp peak (Figure 2F). Any additional peaks observed in mutant samples were therefore considered indels (Supplementary Table S11; Supplementary Figure S2). For all lines, overall indel frequency closely matched NGS. The largest difference observed was in N3a, which showed 8.3% lower indel frequency with CE than with NGS-CS. Estimated indel size was also similar, with only N11a showing a difference. For N11a NGS revealed 3, 7, and 14 bp deletions, where CE predicted 2, 6, and 13 bp deletions (Figures 2G,H; Table 1).

In comparison, the *NAC108* sgRNA668 amplicon exhibited a single WT peak at 206 bp and was accurate within 1.8% for all lines in terms of overall non-WT reads/peaks frequencies compared to NGS (Figure 2I; Table 1). In line N11a, sgRNA668 CE predicted indel sizes of +1 - 1, and – 8, where NGS predicted +1 - 1, and – 7. However, this was associated with the indel size being rounded up from 7.56 to 8.0 when estimated for CE (Figures 2J,K; Table 1; Supplementary Table S9).

The *LG1* WT also exhibited a single peak at 239 bp (Figure 2A; Supplementary Table S12) and the amplicon contained both *LG1* sgRNA targets. For this gene target, if an indel occurred at both sgRNA sites within a single allele, it is presented as a single peak, rather than two separate peaks. Due to this, results were more variable than observed with the *NAC108* targets and can be most closely compared to the CS NGS analysis (Table 2). For example, T44a showed a deletion of 22 bp in CE. This closely matched CS. As expected, it differed from CA, which showed two separate indels of – 1 and – 21 at the sgRNA41 and sgRNA25 sites, respectively (Figures 2B–E; Table 2). Similar observations were found in other lines which contained edits at both sites (Supplementary Figure S3). Also, interesting to note that CE detected large deletions in the lg*1* lines (54 bp in L11a-b; Figure 5A), consistent with CS, which were not detected using the CA software (Table 2). Adding the large deletions increased the overall editing frequency estimated for those lines with CE or CS compared to CA (Table 2).

#### 3.3.2 CE indel estimation accuracy with multiple WT peaks (*tgd5* lines)

For *TGD5* sgRNA73, two WT peaks were observed: a primary peak at 227 bp and a smaller secondary peak at 226 bp (Figure 2L). An amplicon matching the smaller second peak was not observed in either NGS analysis, possibly due to mismatches with the conserved sequence and indicator sequences used in CS and CA, respectively. Due to the presence of a second peak, any peaks matching that size were considered WT peaks in mutant *tgd5* lines (Supplementary Figure S4). This led to an underestimation of overall indel frequencies for all lines, ranging between 1.4% and 22.2% (Table 3; Supplementary Table S13). For lines T32, T41, and T53 this was mostly due to 1 bp deletions that were not being called accurately, as they matched the second WT peak (226 bp; Figure 2L; Table 3). In addition, a 2 bp insertion was correctly predicted in T34. However, a +2 bp peak was not observed in T82, where NGS showed a 2 bp insertion (Figure 2M–N; Table 3). Apart from these discrepancies, all remaining indel size estimation remained accurate (Table 3).

Similarly, the *TGD5* sgRNA24 target amplicon presented displayed three WT peaks. A main WT peak was observed at 227 bp, with additional smaller peaks at 226 and 231 bp (Figure 2O). In comparison to the sgRNA73 CE, this had a smaller impact on the overall indel frequencies observed due to reduced occurrence of 1 bp deletions (Table 4). The largest observed difference from NGS was 13.3% in T208, which had a 37.7% frequency of non-WT peaks in CE and a 50.9% frequency of non-WT reads in CS NGS (Table 4). This was again due to a missing 1 bp deletion. CE indel size estimates at sgRNA24 were observed to vary by 1-2 bp for a few of the longer deletions compared to NGS predictions. For example, in T32, CA indicated 17 and 35 bp deletions, CS indicated 17, 18, and 35 bp deletion, and CE called as 18, 19 and 36 bp deletions. However, when combined, the estimated total frequency of deletions matches for all of the analyses (Table 4; Supplementary Figure S5).

### 3.4 Cas9 RNP assay

#### 3.4.1 Cas9 RNP assay optimization

**FIGURE 3:**
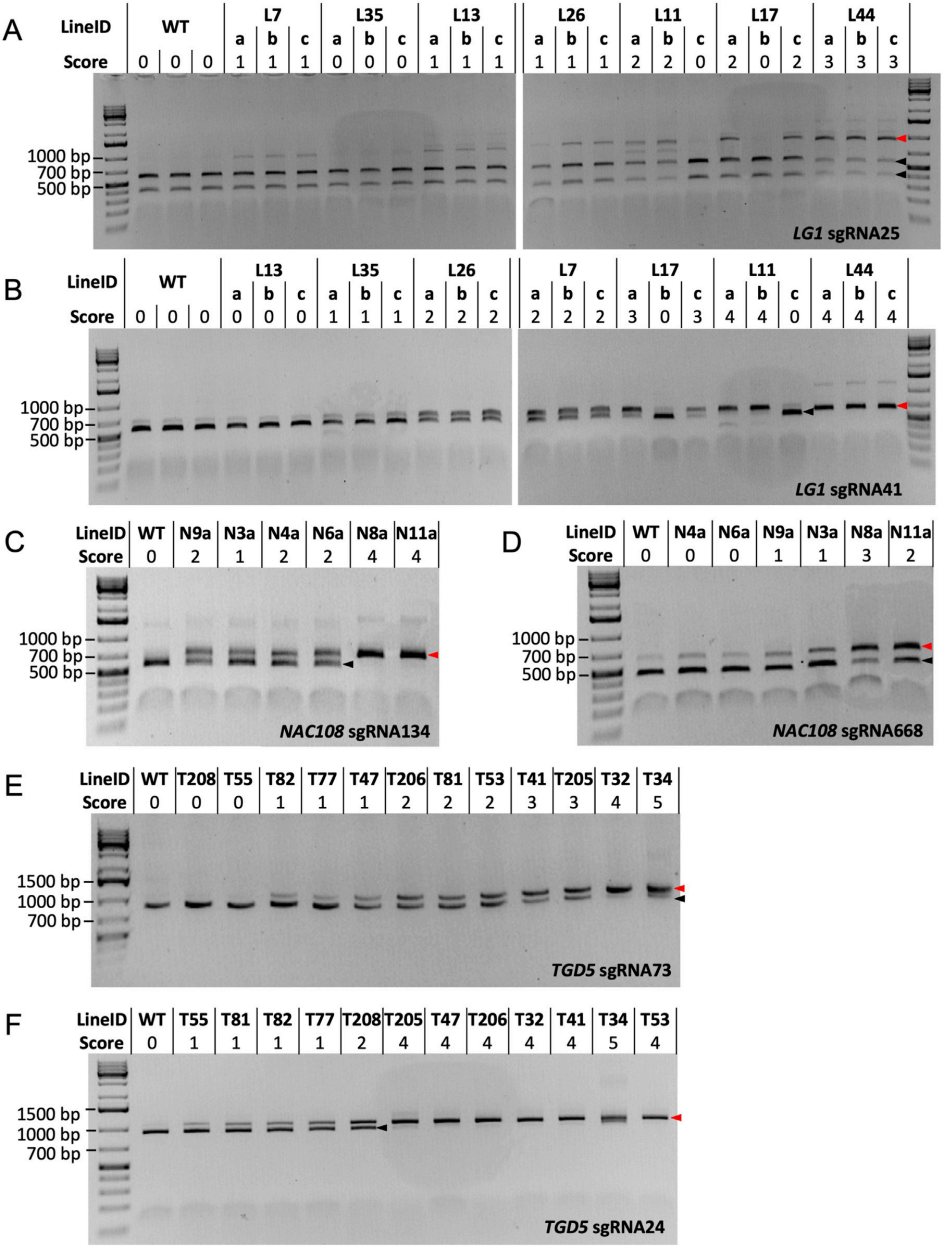
Cas9 RNP assay results. Black arrows indicate the size of cleaved amplicons (WT alleles), where red arrows indicate the size of un-cleaved amplicons (mutated alleles). **(A)** RNP assay results for *LG1* sgRNA25. WT DNA = ∼414 bp and ∼541 bp, mutated DNA = ∼955 bp; **(B)** RNP assay results for *LG1* sgRNA41. WT DNA = ∼541 bp, mutated DNA = ∼647 bp; **(C)** RNP assay results for *NAC108* sgRNA134. WT DNA = ∼540 bp, mutated DNA = ∼674 bp; **(D)** RNP assay results for *NAC108* sgRNA668. WT DNA = ∼455 bp, mutated DNA = ∼614 bp; **(E)** RNP assay results for *TGD5* sgRNA73. WT DNA = ∼852 bp, mutated DNA = ∼1,048 bp; **(F)** RNP assay results for *TGD5* sgRNA24. WT DNA = ∼927 bp, mutated DNA = ∼1,048 bp.

A combination of 200 ng Cas9 protein, 100 ng of sgRNA/scaffold, and 100 ng target DNA PCR product was found to be optimal for all *LG1* and *TGD5* RNP assays, allowing complete (or near complete) cleavage to be observed in WT samples (Figure 3). However, when following this protocol, *NAC108* sgRNA668 failed to generate complete cleavage of the WT amplicon. Different concentrations of the Cas9 protein, sgRNA/scaffold, and template DNA were therefore compared to optimize the reaction. Increasing sgRNA/scaffold or template DNA above 100 ng resulted in reaction failure, regardless of the Cas9 protein concentration used. However, a Cas9 protein concentration of 300 ng, with 100 ng of sgRNA/scaffold and 100 ng WT target DNA, allowed complete cleavage to be observed for *NAC108* sgRNA134 (band ∼540 bp), and near complete cleavage at sgRNA668 (band ∼455 bp; Figures 3C,D). In comparison, for *LG1* and *TGD5* assays, increasing Cas9 protein concentration to 300 ng resulted in reaction failure. The quality of results also decreased if target DNA amplicons were subject to multiple freeze/thaw cycles (data not included).

While the amplicon used for the sgRNA41 RNP assay also contained the sgRNA25 site, the small difference in amplicon size between sgRNA25 cleaved (WT; 584 bp) and un-cleaved (mutated; 647 bp) DNA for this amplicon made mutation identification challenging (Supplementary Figure S1). Therefore, the *LG1* sgRNA25 RNP assay was redesigned to contain a larger target DNA amplicon, resulting in two products of distinct size for cleaved WT DNA (∼414 bp and ∼541 bp) and a single ∼955 bp product when un-cleaved (mutant DNA; Figure 3A).

#### 3.4.2 *LG1* mutant confirmation via sgRNA25 and sgRNA41 RNP assays

For the *LG1* sgRNA25 RNP assay, WT responded with two products of ∼414 bp and ∼541 bp (score 0). Interestingly, L7a-c scored 1, with faint bands appearing at ∼955 bp, suggesting un-cleaved product. This is in direct contrast to the NGS results for these lines, which show no indels at the sgRNA25 site (Table 2). In comparison, scores for all other lg*1* lines corresponded to the NGS edit frequencies at the sgRNA25 site, including both L11 and L17, which showed potential chimerism with partial cleavage in vegetative progenies L11a, L11b, L17a, and L17c (score 2) vs complete cleavage in L11c and L17b (score 0; Figure 3A).

In the *LG1* sgRNA41 RNP assay, WT amplicons resulted in a cleaved product which presented as a band of ∼541 bp following gel electrophoresis. Complete cleavage could not be achieved in this assay due to known SNPs within the sgRNA sequence in 4/40 *LG1* alleles (Brant et al., 2024), so a very faint un-cleaved product (∼647 bp) was also visible in WT. This was scored as 0 (Figure 3B). Similar to the *LG1* sgRNA25 RNP assay, chimerism was also observed within the L11 and L17 progenies here. Scores for L13a-c, L35a-c, L17a,c, and L44a-c matched with corresponding NGS data (0, 1, 3, 4, and 4 for 0%, ∼10%, ∼55%, and ∼95% indel frequency, respectively; Table 2). However, L26a-c and L11a-b were scored 2 and 4, which were slight overestimations in comparison to the NGS derived indel frequencies (∼34% and ∼80%, respectively; Figure 3B; Table 2).

#### 3.4.3 *TGD5* mutant confirmation via sgRNA73 and sgRNA24 RNP assays

In the *TGD5* sgRNA24 RNP assay, the WT sample contained a single cleaved product (∼927 bp) and was scored 0. T55, T81, and T82 were all scored 1, with both the cleaved and un-cleaved product (∼1,048 bp) present (Figure 3F). This matched with the NGS indel frequencies observed in these lines (∼10.8%, ∼13.0%, and 18.1%, respectively; Table 4). While T77 was also scored 1, the NGS indicated ∼45.2% indel frequency, suggesting using the RNP assay alone would result in an under estimation of indel frequency (Figure 3F; Table 4). Interesting, line T208, which had an increase in indel frequency of only ∼5% over T77, was scored 2 in the RNP assay (Figure 3F). All other lines were scored 4-5, matching with observed NGS indel frequencies of 90%–100% (Figure 3F; Table 4).

In the *TGD5* sgRNA73 RNP assay, WT was indicated by a single cleaved product of ∼852 bp (score 0; Figure 3E). This was also observed in lines T208 and T55, which matched with the 0% indel frequencies noted in both lines using NGS (Figure 3E; Table 3). Interestingly, line T81 scored 1, showing a faint un-cleaved product at ∼1,048 bp despite having an indel frequency as low as 2.7% (Table 3). RNP assay scores for all other *tgd5* lines in this assay fell within expected ranges when considering NGS indel frequencies (Figure 3E; Table 3).

#### 3.4.4 *NAC108* mutant confirmation via sgRNA134 and sgRNA668 RNP assays

In the *NAC108* sgRNA134 RNP assay, a single cleaved product of ∼540 bp was observed in WT (score 0). In comparison, a single band of ∼674 bp was observed for lines N8a and N11a (score 4; Figure 3C), matching with the NGS data showing 100% indel frequency at this site in both lines (Table 1). Frequencies predicted for N3a (score 1) and N6a (score 2) also matched with available NGS data (∼30% and 40%, respectively). However, as was observed in the *LG1* sgRNA41 RNP assay, lines N4a and N9a both scored 2, which was an overestimation of their indel frequencies (35% for both; Figure 3C; Table 1).

In the *NAC108* sgRNA668 RNP assay, as complete cleavage was not obtained, a strong signal at ∼455 bp was observed in WT, representing the cleaved product, alongside a faint signal at ∼614 bp, for the un-cleaved product (Figure 3D). This was scored 0 to represent no mutations. Only lines N9a (score 1), N3a (score 1), N8a (score 3), and N11a (score 2) were distinguishable from WT, showing signals for both cleaved and un-cleaved products (∼455 bp and ∼614 bp; Figure 3D). While N3a and N11a match with the corresponding NGS results (Table 1), mutation frequency at N8a was overestimated with the RNP assay (NGS = ∼50%). N4a was considered WT (score 0) on the RNP gel (Figure 3D) when analyzed using ImageJ, despite containing a 8.8% indel frequency (Table 1).

### 3.5 High resolution melt analysis (HRMA)

#### 3.5.1 Primer selection for HRMA

As HRMA requires specific amplification of a short region surrounding the target, multiple primer pairs were evaluated to obtain the pair most likely to obtain accurate results. Initially, *LG1* HRMA primers were designed with the aim of analyzing each sgRNA target individually (l2541–1, l2541–2, l2541-3; Supplementary Table S4). However, with the short distance between the sgRNA sites (20 bp) and the restrictions on HRMA amplicon length and conserved sequences, one primer within each pair was homologous to the non-target sgRNA site. As mutations are observed at both sites in most lines, this led to biased amplification. Primer pairs were therefore re-designed to cover both target sites simultaneously, with l2541-5 selected for HRMA (130 bp amplicon; Supplementary Table S4).

Due to the short exon sequences observed in *TGD5,* all HRMA primers within this gene had to be designed to anneal to introns flanking the short target exons. For *TGD5* sgRNA73, two pairs were assessed (t73-1 and t73-2). While both pairs incited only a single melt peak, t73-2 exhibited better amplification (107 bp amplicon) and was therefore selected for use in HRMA (Supplementary Table S4). Four primer pairs were assessed for *TGD5* sgRNA24, with two combinations (t24-1 and t24-4) showing unspecific amplification and a third (t24-3) resulting in only a low level of amplification (Supplementary Table S4). Primer pair t24-2 was selected (101 bp amplicon). However, the reverse primer used in this pair was designed in a region not conserved between all copies of the *TGD5* gene indicated in the *Saccharum officinarum* LA purple genome and may therefore have incited bias within the assay.

For *NAC108* sgRNA134 three pairs were tested, one of which showed additional melt peaks in the WT samples (n134-1). While both of the other two pairs showed specific amplification and produced the same sized amplicon (132 bp), n134-2 showed a higher melt peak and was therefore selected for HRMA. For sgRNA668, while n688-1 produced a shorter amplicon (90 bp), it showed additional melt peaks for WT, suggesting unspecific amplification (Supplementary Table S4). Therefore n688-2 was selected (102 bp).

#### 3.5.2 Identification of mutant lines using HRMA

**FIGURE 4:**
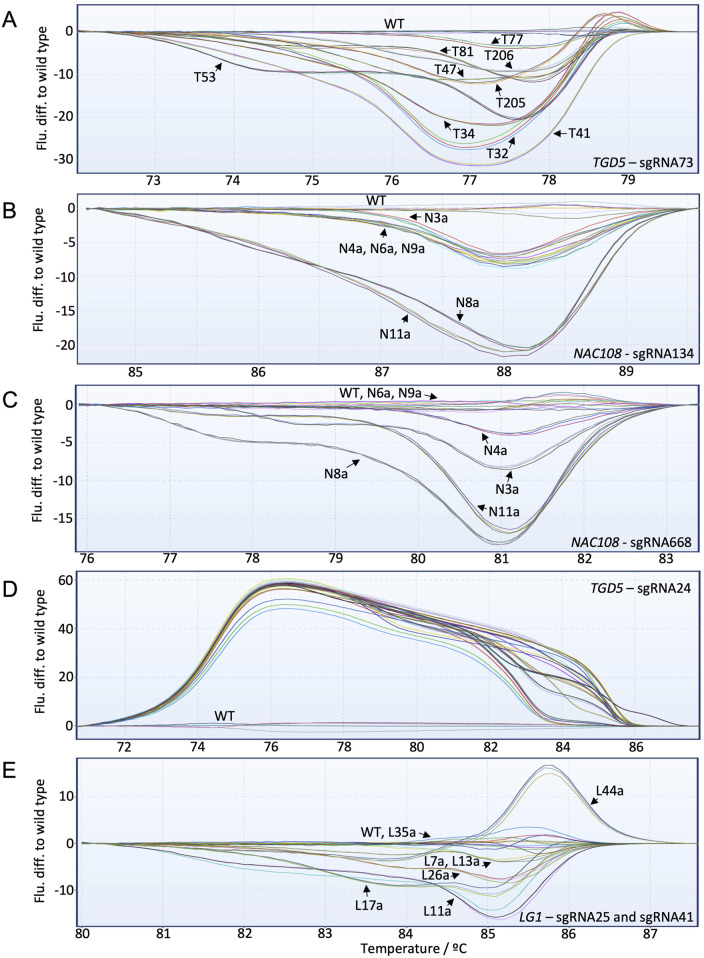
High-resolution melt analysis (HRMA) fluorescence difference to WT graphs for all targets included in the study. Individual lines are labeled with black arrows. **(A)** *TGD5* sgRNA73 HRMA assay results; **(B)** *NAC108* sgRNA134 HRMA assay results; **(C)** *NAC108* sgRNA668 HRMA assay results; **(D)** *TGD5* sgRNA24 HRMA assay results; **(E)** *LG1* HRMA assay results.

For the *TGD5* sgRNA73 site, HRMA distinguished clearly between each mutant line (Figure 4A; Supplementary Figure S6). This includes line T77, which showed ∼16.3% editing with NGS. In general, the fluorescence difference from WT increased as the number of non-WT reads increased. However, some lines deviated from this trend. For example, T41 showed only ∼57% non-WT reads with NGS but had the greatest fluorescence difference to WT with HRMA (Figure 4A; Table 3).

For both *NAC108* target sites all mutant lines were distinguishable from WT using HRMA, with fluorescence difference from WT increasing as frequency of non-WT reads increased (Figures 4B,C). Line N4a, which had the lowest number of NGS reads with variation to WT (8.6%) according to CA showed a clear difference to WT in HRMA at sgRNA668 (Table 1; Supplementary Figure S7).

For the *TGD5* sgRNA24 site, while all mutant lines were clearly distinguishable from WT. However, maximum fluorescence difference from WT was similar for all lines (between 50–60) regardless of mutation frequency observed with NGS (Figure 4D; Table 4; Supplementary Figure S8). Most lg*1* lines were distinguishable from WT with HRMA. However, L35a, which showed ∼10% non-WT reads with NGS, appeared as WT (Figure 4E; Table 2). In addition, maximum fluorescence difference from WT did not increase as frequency of non-WT reads increased with the *LG1* assay, with L7a and L13a both showed a maximum fluorescence difference between 3-5 yet having a ∼20% difference in NGS non-WT read frequency (Figure 4E; Table 2). High similarity was observed between progenies which did not show chimerism (Supplementary Figure S9).

### 3.6 Identification of chimerism within lg*1* mutant lines

**FIGURE 5:**
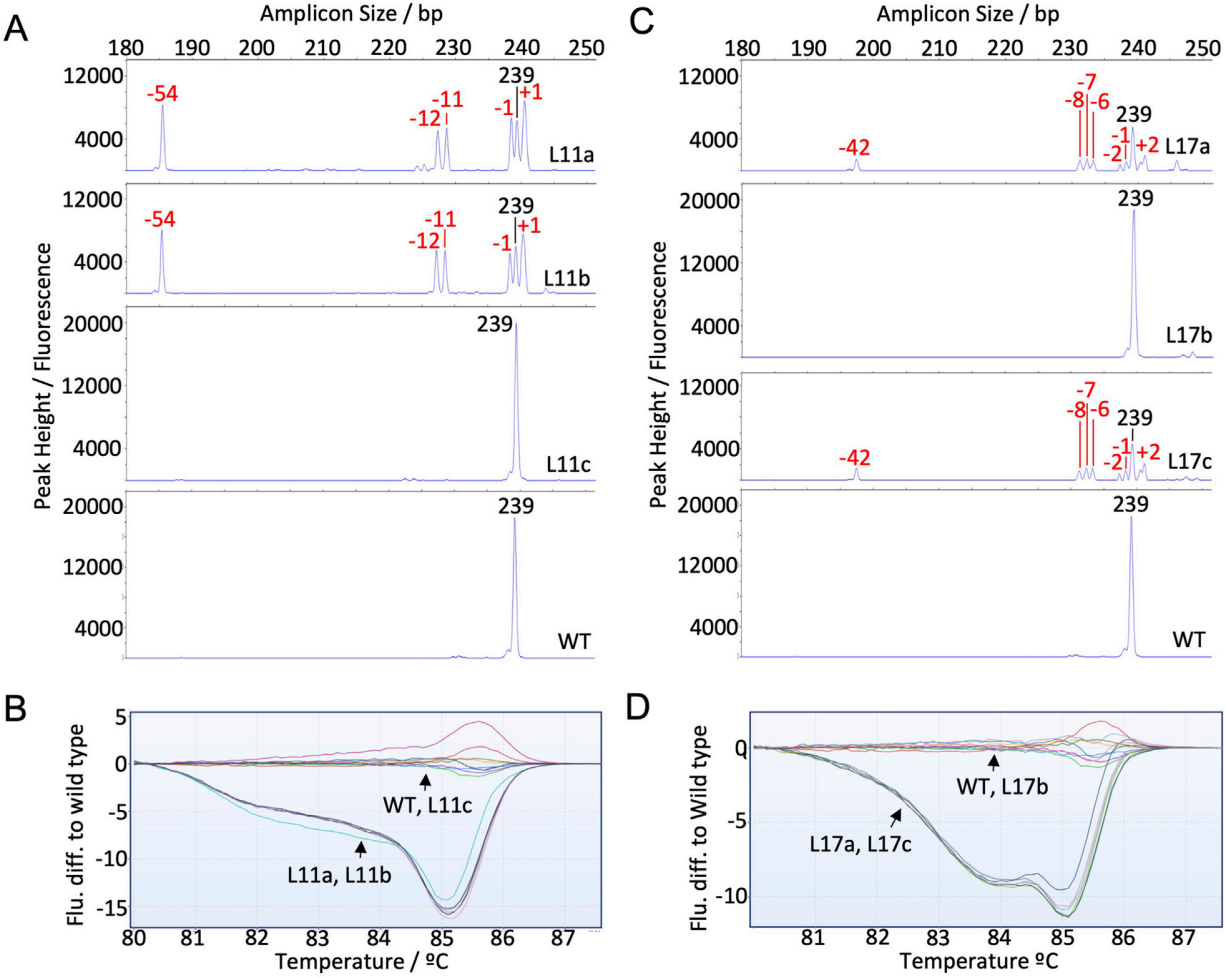
Capillary electrophoresis (CE) peak fluorescence graphs and high-resolution melt analysis (HRMA) results for lines showing chimerism. The size of WT peaks/amplicons is labeled by bp in black, where indels are labeled by size in red on CE graphs. **(A)** *LG1* CE for lines L11a, L11b, and L11c; **(B)** HRMA fluorescence difference to WT for L11a, L11b, L11c; **(C)** *LG1* CE for lines L17a, L17b, and L17c; **(D)** HRMA fluorescence difference to WT for L17a, L17b, L17c.

For lg*1* mutants, three vegetative progenies were tested per mutant background with each of the methods to determine the potential of the methods for identifying chimeric events. Two chimeric events were identified with NGS from the L11 and L17 backgrounds (Brant et al., 2024). From L11, L11a-b both showed ∼79% non-WT reads, where L11c displayed a WT genotype (Table 2). This was detected with all three methods (Figures 3A,B; Figures 5A,B). Similarly, from L17, L17a and L17c both showed ∼73% non-WT reads, where L17b presented as WT (Table 2). This was also clearly distinguishable with all three methods (Figures 3A,B; Figures 5C,D).

### 3.7 Comparison of costs associated with CE, HRMA, Cas9 RNP assays, and NGS

Estimated costs associated with running each assay are presented in Supplementary Table S6. CE presented as the least expensive assay, with a per sample cost estimated at $3.82, which was ∼6% of the cost of NGS (∼$64 per sample). This was followed by HRMA, which despite requiring samples to be run in triplicate, was estimated to cost $4.44 per sample. Cas9 RNP assay was the most expensive non-NGS assay ($14.87 per sample). However, the Cas9 RNP assay was still around 75% less expensive than NGS.

## 4 Discussion

Sugarcane has the most complex of all crop genomes (2n = 100–130), with any given loci expected to present at least 10–12 homo (eo)logous copies throughout the genome (de Setta et al., 2014; Souza et al., 2019). To obtain a loss of function phenotype in sugarcane with genome editing, co-editing of all or a high proportion of the expressed copies/alleles is therefore required (May et al., 2023a). Confirming mutation success is most commonly undertaken using short read NGS, which allows an estimation of the extent of co-editing based on read frequency (Eid et al., 2021). However, thorough evaluation of the co-editing of allelic variants requires either Sanger sequencing of cloned amplicons or use of long read NGS platforms (Oz et al., 2021). As generation of a large number of transformed plants is typically required for identification of lines with the desired level of co-editing, these genotyping approaches are expensive (Liang et al., 2018; Brant et al., 2024). Additionally, the most frequent indels created by NHEJ are small, 1 bp variations (Zhu et al., 2017). While such mutations can create a loss of function, they are challenging to detect with many non-sequencing methods (Pan et al., 2016; Ren et al., 2016; Zhu et al., 2017). Therefore, to optimize identification of gene edited sugarcane or other polyploids, sensitive, cost-effective, and high throughput methods for genotyping are desired. Here, we demonstrated successful application of three genotyping methods for sugarcane mutant screening: HRMA, Cas9 RNP assays, and CE.

While all three methods were able to distinguish mutant lines from WT, CE provided the most comprehensive output, indicating overall mutation frequency, individual indel size, and individual indel frequency. CE offered the closest comparison to NGS data, with straightforward assay design at a fraction of the NGS cost. As several life science service companies offer CE, completing the analysis was as simple as running PCR, diluting products in a buffer, and shipping the samples to the selected service. While optimization may be required to ensure CE peaks aren’t over saturated, these issues can typically be resolved by adjusting DNA concentration, injection speed, and/or run time (Andersen et al., 2003). We previously demonstrated the utility of CE for analysis of large, targeted mutations induced with TALENs in sugarcane (Jung and Altpeter, 2016; Kannan et al., 2018). The largest mutation observed at the CRISPR target sites was 64 bp (*TGD5* sgRNA24), and this was accurately identified using CE. Additionally, CE identified large CRISPR-mediated indels (>40 bp) at one of the targets (*LG1*) which were missed by NGS analyses using the CA software. CE was also able to achieve the high resolution necessary to identify 1 bp indels, even when present only at a low frequency in sugarcane. This was critically important considering that the most common indels observed across the six sgRNA target sites were 1 bp insertions or deletions, as described earlier for other crops (Pan et al., 2016; Ren et al., 2016; Zhu et al., 2017).

In wheat, which contains three subgenomes (A, B, and D), it has previously been demonstrated that CE can identify indels of 1-3 bp at high frequencies (Okada et al., 2019). CE was utilized in a high through-put assay in tetraploid potato, enabling identification of 129 mutant lines, which were shown to correlate well to sequencing results (Andersson et al., 2017). In comparison to both of these studies, the sugarcane targets analyzed were more genetically complex. For example, in wheat and potato, three and four gene copies were present for each target, respectively (Andersson et al., 2017; Okada et al., 2019). For *LG1, >*40 copies were present (Brant et al., 2024). Regardless of this challenge, CE was still able to accurately estimate indel frequency and size.

Taking this a step further, Schernthaner et al. (2022) recently published a method which utilized SNPs to design allele specific primers for three gene copies in hexaploid wheat, allowing each amplicon to subsequently be reamplified with differentially labeled universal primers and mixed into one CE sample. This method allowed knowledge to be gained regarding which of the three copies contained indels of which sizes (Schernthaner et al., 2022). This could be beneficial in sugarcane for gathering knowledge on a particular allele/copy of interest, and could be utilized for gene copies with large indels that present as different sized amplicons, as this would provide distinct peak patterns. However, this approach is limited by the number of available fluorescent labels.

An important factor to note with all of the assays is that sgRNA design needs to consider the downstream genotyping analysis. For example, for CE, amplicons between 200 and 260 bp were required to accurately compare to NGS. For one of our target genes (*TGD5*), due to the presence of short exons, primers had to be designed which annealed to introns. As introns typically show lower sequence conservation than coding regions, this contributed to the generation of a small fraction of WT PCR amplicons with size variations, complicating the CE analysis by introducing additional small peaks flanking the main WT peak (Garsmeur et al., 2011). However, where possible, this problem can be overcome by targeting sgRNAs to larger exons.

HRMA becomes less specific as amplicon length increases, with amplicons smaller than 100 bp being most desirable to detect 1 bp mutations (Zischewski et al., 2017). Previously, Mirajkar Shriram et al. (2018) demonstrated HRMA in sugarcane for identifying radiation-induced mutants using large amplicons to isolate only lines containing large, high frequency mutations. While we obtained a higher specificity than this, challenges were still observed. In *T. aestivum* it has been reported that HRMA can distinguish between mutant lines containing 1 bp indels or SNPs*,* both with and without use of a nested PCR approach to segregate gene copies prior to analysis (Dong et al., 2009; Botticella et al., 2011). In *Arabidopsis*, HRMA was able to distinguish samples containing <5% of mutant DNA (1 bp indel) pooled with WT DNA (Denbow et al., 2017). In theory, this should allow distinguishing polyploid lines with low frequency mutations from WT lines. However, the specificity of HRMA for identifying low frequency mutations appears to vary between target sites in sugarcane, as does its capacity to indicate mutation frequency. For both the *NAC108* assays and the *TGD5* sgRNA73 assay, a trend between fluorescence difference to WT and mutation frequency was observed. However, the other targets did not show this trend. The variable specificity observed is likely a result of primer sequence and location (intron vs exon), melt temperature, and/or the number of SNPs present in and G/C content of the amplicon (Dong et al., 2009; Thomas et al., 2014). As many of these factors are independent, yet have a combinatory affect, it is challenging to assign responsibility for the variation in specificity at different targets to a single factor. For example, the *LG1* HRMA was unable to distinguish an ∼10% mutated line from WT. This may in part be explained by the size of amplicon used (130 bp), which was larger than the recommended 100 bp in order to co-amplify two neighboring sgRNA sites. However, the mutated line (L35) contained an 8 bp deletion, so should have required lower resolution to distinguish it from WT (Thomas et al., 2014). Adding to this, the *NAC108* sgRNA134 assay also used a larger amplicon and was able to more clearly indicate a trend related to mutation frequency, suggesting that more factors than just amplicon size were affecting the outcome.

In contrast to *Arabidopsis* and other dicots, sugarcane has extensive allelic variation in the target amplicons and the abundance of SNPs reduces the utility of HRMA for detecting events with low indel frequency in sugarcane and other polyploid species. Variability was observed between runs of WT samples, a phenomena which was also observed in *T. aestivum* (Dong et al., 2009). This could be exasperated if amplicons of different sizes are present within the sample due to large indels, suggesting assay design and optimization need to be taken into consideration at the time of sgRNA site selection to mitigate these issues where possible (Zischewski et al., 2017).

While assay design can be challenging, once optimized for a target, HRMA remained the quickest, most high-throughput method of confirming mutation presence, with each run taking <2 h. The cost per sample for HRMA is also low despite running each sample in triplicate. However, HRMA does require specific instruments and is limited to only identifying mutations smaller than the amplicon size (<90 bp for *NAC108* sgRNA668) (Thomas et al., 2014). This makes it a good option for rapid mutant screening in sugarcane if instruments are available and quantifiable information regarding mutation frequency and size are not required. Interestingly, it was demonstrated by Gady et al. (2009) that a similar throughput was obtained when screening mutated *S*. *lycopersicum* populations using either CE or HRMA.

The decision to run samples in triplicate only for HRMA was based on the tool’s high sensitivity to sequence variation, which led to variation being observed between technical replicates of WT samples. This was likely due to the presence of SNPs outside of the sgRNA sites in the target amplicons, combined with potential amplification bias occurring during the PCR steps (Gottlieb, 2003; Dong et al., 2009). As HRMA difference graphs use WT as a baseline, even minor variation between runs of a WT or mutant sample could have implications on the predictive power of the tool (Han et al., 2012). In comparison, the results of CE, Cas9 *in vitro* assays, and NGS, is not affected by SNPs outside of the sgRNA sequence, so the running samples in triplicate was considered unnecessary for these tools.

One limitation observed with CE is its lack of ability to indicate the presence of base substitutions (Zischewski et al., 2017; Lomov et al., 2019). This is something that has previously been achieved with HRMA in tetraploid alfalfa (*M. sativa)*, and could be explored further in sugarcane (Han et al., 2012). However, with the challenges observed in identifying low frequency indels using HRMA in this study, it is likely that the application of HRMA for this purpose will be restricted by the efficiency of each individual target/assay, decreasing its applicability.

In comparison to CE and HRMA, Cas9 RNP assay was the most laborious assay considered, requiring multiple rounds of PCR, an RNA synthesis and cleaning step, and an overnight incubation. On average this resulted in assays requiring ∼1 week to set up. However, RNA synthesis and cleaning only needed to be completed once per target, and thousands of reactions could be completed with the sgRNA produced. This decreases the time for subsequent screening at that site to ∼2 days. In wheat, this was also shortened by using a 3–4 h incubation step, instead of overnight (Liang et al., 2018; Gupta and Li, 2021). However, that was found to be ineffective when complete cleavage was required with sugarcane DNA. Completing multiple reactions with each synthesized sgRNA would also decrease the cost per sample, which was estimated based on the idea that RNA would need to be synthesized for each target.

Cas9 RNP assays fell between CE and HRMA in terms of information output. While no information could be gathered on indel size, RNP assays could be scored to provide an indication of mutation frequency, making the assay semi-quantitative. This makes it superior to HRMA in this instance. The RNP assay for *TGD5* sgRNA73 was able to distinguish a line with a mutation frequency of ∼3% (T82), which neither HRMA nor CE were sensitive enough to identify. The RNP assay is also not negatively affected by variations in amplicon sizes due to the presence of WT indels in different alleles outside the sgRNA site, as the assay result only considers the sgRNA site. However, similar to HRMA, the efficiency of the RNP assay varied between targets. Unlike HRMA, this is likely due to the sgRNA efficiency, so could be mitigated by completing a Cas9 *in vitro* assay whilst selecting sgRNA sites prior to transformation, which would allow simultaneous confirmation of sgRNA and assay efficacy (Bente et al., 2020). In comparison to the current most widely used method for sugarcane CRISPR mutant screening (CAPS assays), the Cas9 RNP assays was semi-quantifiable through a scoring system, which has yet to be demonstrated with CAPS in sugarcane. They also provide more flexibility for sgRNA design by removing the requirement of the CAPS assay for an enzyme restriction site to overlap the sgRNA site. The RNP assay also does not depend on the affinity of individual restriction enzymes (Eid et al., 2021; Gupta and Li, 2021; Oz et al., 2021).

For many crop species, the incidence of chimerism has posed a continuous hurdle for optimization of genome editing protocols. As transformation is typically followed by adventitious regeneration, transgenic plants do not always arise from a single cell, which can lead to the formation of chimeric events (Malabarba et al., 2021). These events will contain multiple genetically and phenotypically distinct cells/tissues. Similar challenges are observed in screening chimeras as with initial mutant screening, as the same techniques need to be applied. However, to screen for chimeras, multiple tissue samples taken from different leaves/tillers or progeny of the same transgenic event need to be genotyped to ensure uniformity, further increasing costs (Lal et al., 2015).

In allotetraploid tobacco (*N*. *benthamiana*), out of 174 lines mutated, almost 50% were found to contain mosaic patterns at one or more of the target sites via Sanger sequencing (Jansing et al., 2019). The frequency with which chimeric lines are formed varies between species and protocol, with species such as poplar (*Populus* spp.) and apple (*Malus* spp.) showing particularly high frequencies (90% and >85%, respectively) (Charrier et al., 2019; Ding et al., 2020). While chimerism was observed in early reports of sugarcane transformation, its incidence appears to be low, partially due to having a well-defined transformation and selective system (Bower and Birch, 1992; Gambley et al., 1993; Mayavan et al., 2015). This is the first report confirming chimeric sugarcane mutants created with SSN technologies. While it was demonstrated that all three methods were able to distinguish the chimeras from progeny analysis, which showed either uniform mutations or a WT genotype, this may not be the case if the chimera contained cells with different mutation frequencies. Besides the results presented here, CE has also recently been demonstrated to successfully identify chimeric events an apomictic, tetraploid bahiagrass (*Paspalum notatum* L.) variety, highlighting its sensitivity in another genetically complex species (May et al., 2023b). However, in comparison, the Cas9 RNP assay would not have been able to distinguish a chimera if one progeny plant was 60% mutated, where the other two were 70%, as they would have scored the same in the assay. A similar limitation may have been observed for some of the HRMA targets. This is an important consideration to make when selecting a method for chimera screening and highlights another benefit of the CE system.

The methods discussed in this paper are not designed to eliminate the need for sequencing entirely, as information on mutation sequence is necessary for many applications. Rather, these tools are intended to optimize the sugarcane genotyping pipeline by narrowing the pool of candidate lines to only those with the desired mutations prior to sequencing. In turn, this minimizes sequencing costs. Considering this, it is important to also contemplate the potential of NGS. NGS provided the deepest level of information and accuracy, and is the only method suitable for precisely calling base substitutions (Sánchez-Gómez et al., 2023). Differences were noted between CS and CA, for example, between their ability to recognize large deletions and provide the same result at each target site. While these differences can be attributed to variations in the parameters used for each analysis, their presence highlights the need to optimize a sugarcane NGS data analysis pipeline, as free tools with standard analysis parameters may not provide an accurate representation of the data (Clevenger et al., 2015; Guzmán-López et al., 2021; Sánchez-Gómez et al., 2023). This may increase the cost of NGS if bioinformatic analysis and optimization need to be outsourced, particularly if each target site requires optimization.

In summary, all three methods discussed are capable of screening mutant sugarcane lines, with varying levels of specificity. CE represents the most comprehensive, cost-effective method investigated, providing information on individual indel frequency and size, with low labor requirements. In terms of quantitative information, this is followed by the Cas9 RNP assay, which can be scored to indicate overall mutation (within ∼25%) with a higher price and labor requirement. HRMA, in comparison, represent the highest throughput assay with low price and labor requirements. However, while it holds the potential to be semi-quantitative, its consistency across different targets is limited and requires substantial optimizations.

## Data Availability

The datasets presented in this study can be found in online repositories. The names of the repository/repositories and accession number(s) can be found below: https://www.ncbi.nlm.nih.gov/, PRJNA963213; https://www.ncbi.nlm.nih.gov/, PRJNA1026135.

## Abbreviations

NGS, Next-Generation Sequencing; CE, capillary electrophoresis; RNP, ribonucleoprotein; HRMA, high-resolution melt analysis; CRISPR, clustered regularly interspaced short palindromic repeats; sgRNA, single guide RNA; SSN, sequence specific nucleases; Cas9, CRISPR-associated protein 9; NHEJ, non-homologous end joining; HDR, homology directed repair; indels, insertions/deletions; PAM, protospacer adjacent motif; *COMT, CAFFEIC ACID O-METHYLTRANSFERASE; MgCH, MG-PROTOPORPHYRIN IX CHELATASE; LG1, LIGULELESS 1*; PCR, polymerase chain reaction; CA, CasAnalyzer; CAPS, cleaved amplified polymorphic sequence; TALENs, transcription activator-like effector nucleases; *NAC108*, *NAC-TRANSCRIPTION FACTOR 108*; *TGD5, TRIGALACTOSYLDIACYLGLYCEROL 5*; bp, base pairs; *npt*II, *neomycin phosphotransferase* II; WT, wild type; SNPs, single nucleotide polymorphisms; CS, conserved sequence; *R*, indicator range; *GV*, mean grey value.
