# Supplementary material for: Comparison of genotyping assays for detection of targeted CRISPR/Cas mutagenesis in highly polyploid sugarcane
Source: Front Genome Ed. 2024 Dec 12;6:1505844. doi: 10.3389/fgeed.2024.1505844 (PMC11669508; doi:10.3389/fgeed.2024.1505844)
Supplement: Supplementary file 1 [file DataSheet1.pdf]

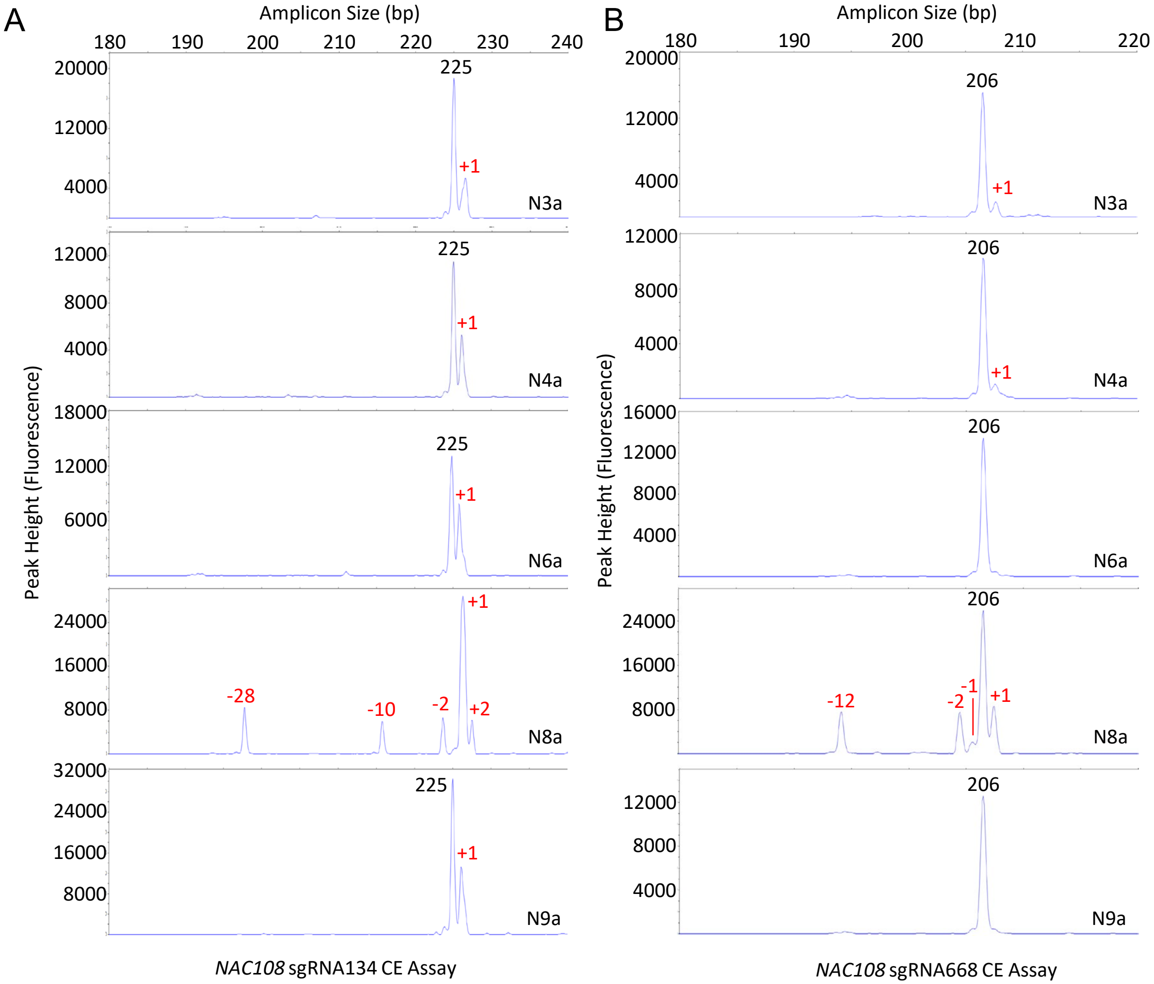

Supplementary Figure 1. Capillary electrophoresis (CE) peak fluorescence graphs. WT peak is labelled in bp in black. Mutant peaks are labeled by indel size in red. A) *nac108* lines analyzed at *NAC108* sgRNA134*;* B) *nac108* lines analyzed at *NAC108* sgRNA668.

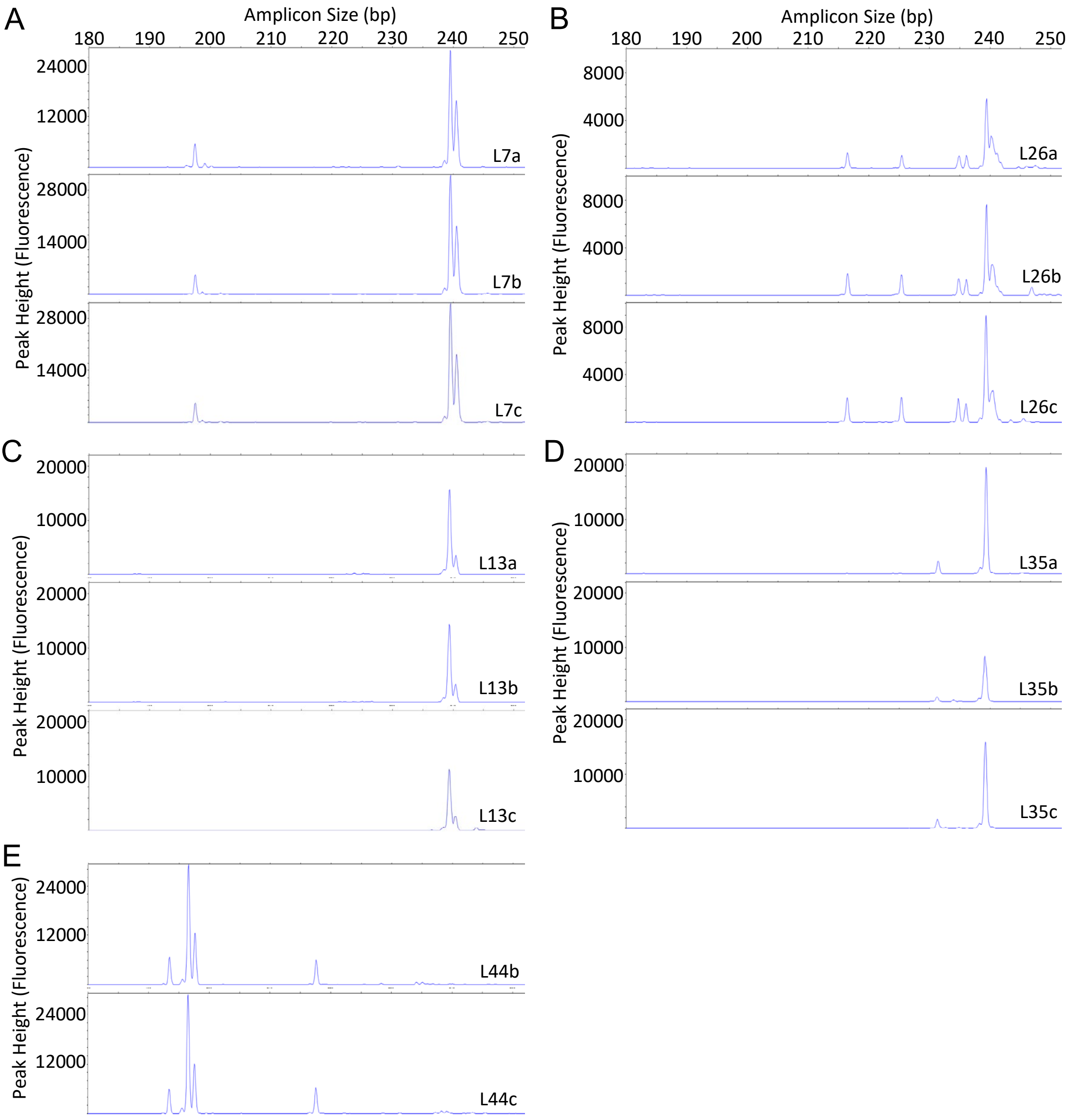

Supplementary Figure 2. Capillary electrophoresis (CE) peak fluorescence graphs for *LG1*. A) Lines L7a, L7b, and L7c; B) Lines L26a, L26b, and L26c; C) Lines L13a, L13b, and L13c; D) Lines L35a, L35b, and L35c; E) Lines L44b and L44c.

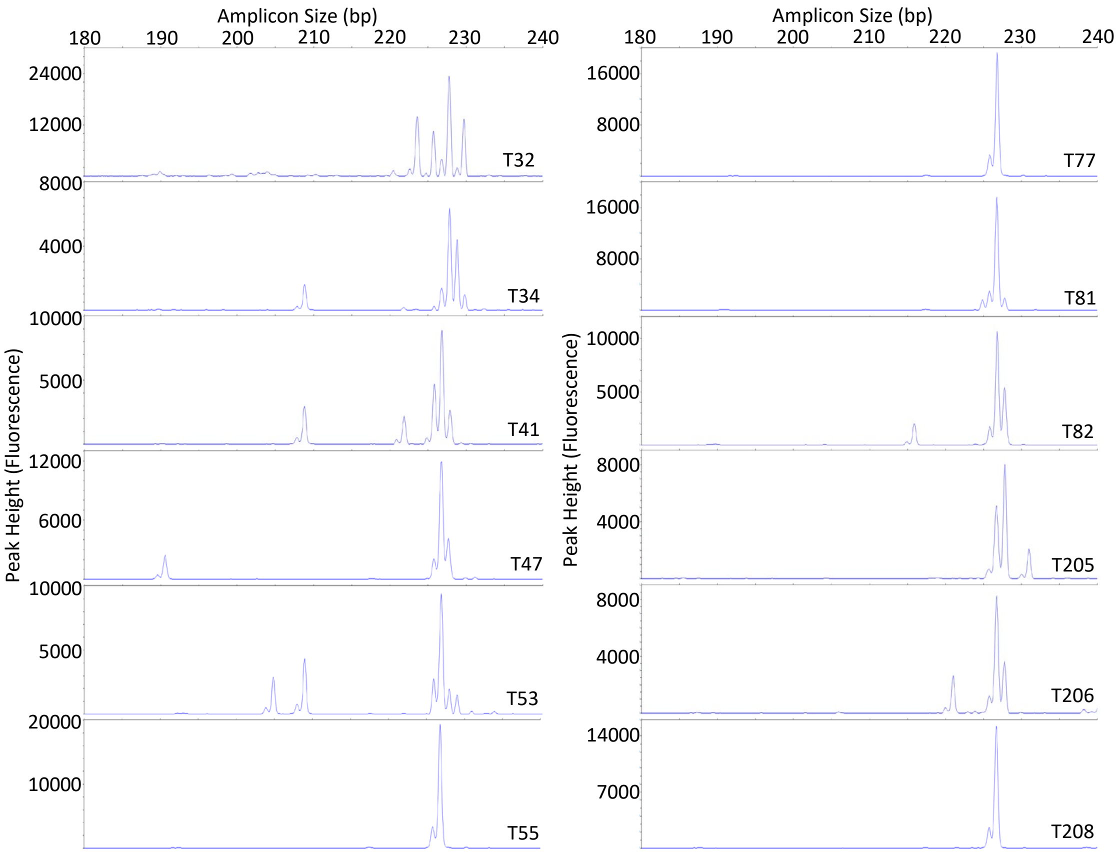

Supplementary Figure 3. Capillary electrophoresis (CE) peak fluorescence graphs for *TGD5* sgRNA73 in *tgd5* lines.

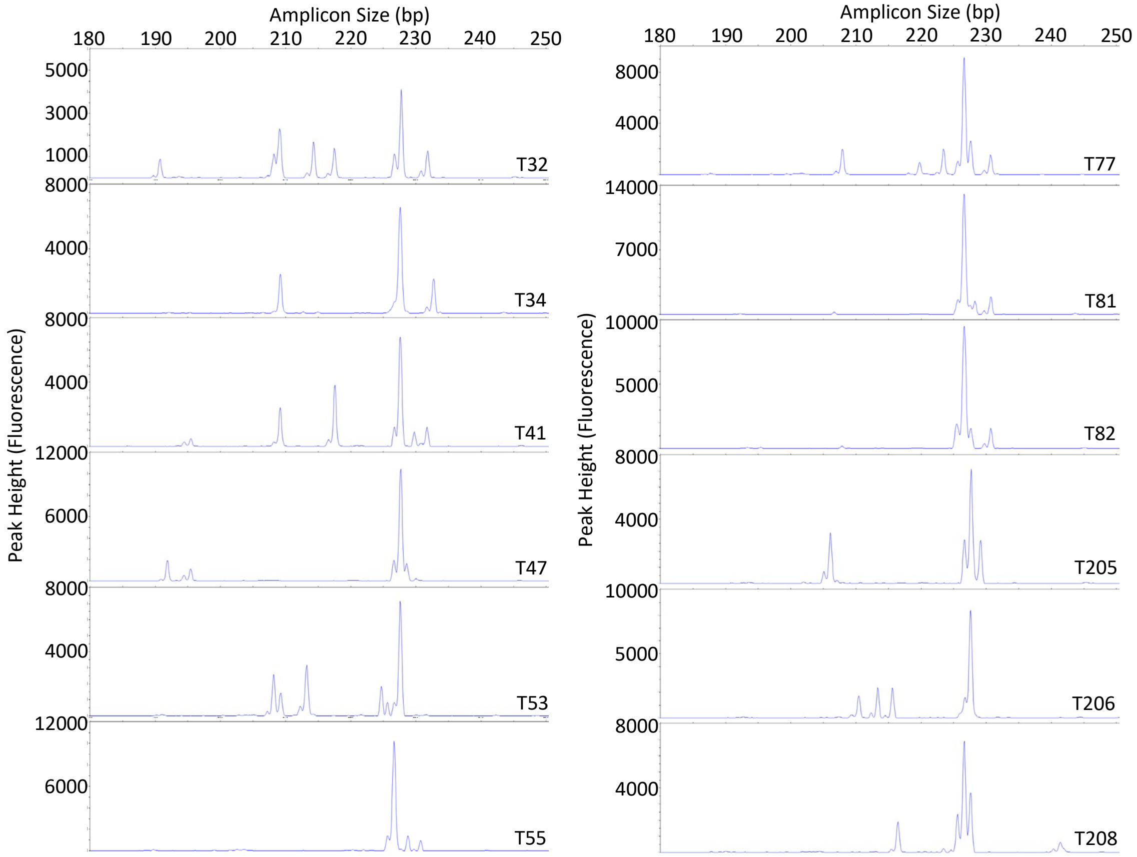

Supplementary Figure 4. Capillary electrophoresis (CE) peak fluorescence graphs for *TGD5* sgRNA24 in *tgd5* lines.

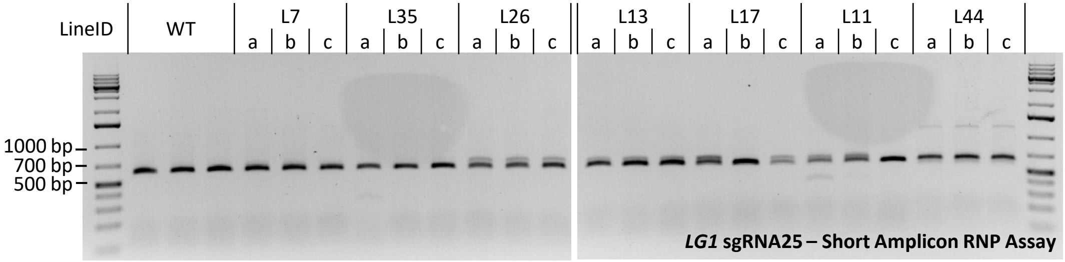

Supplementary Figure 5. Cas9 RNP assay results for *LG1* sgRNA25 short amplicon assay.

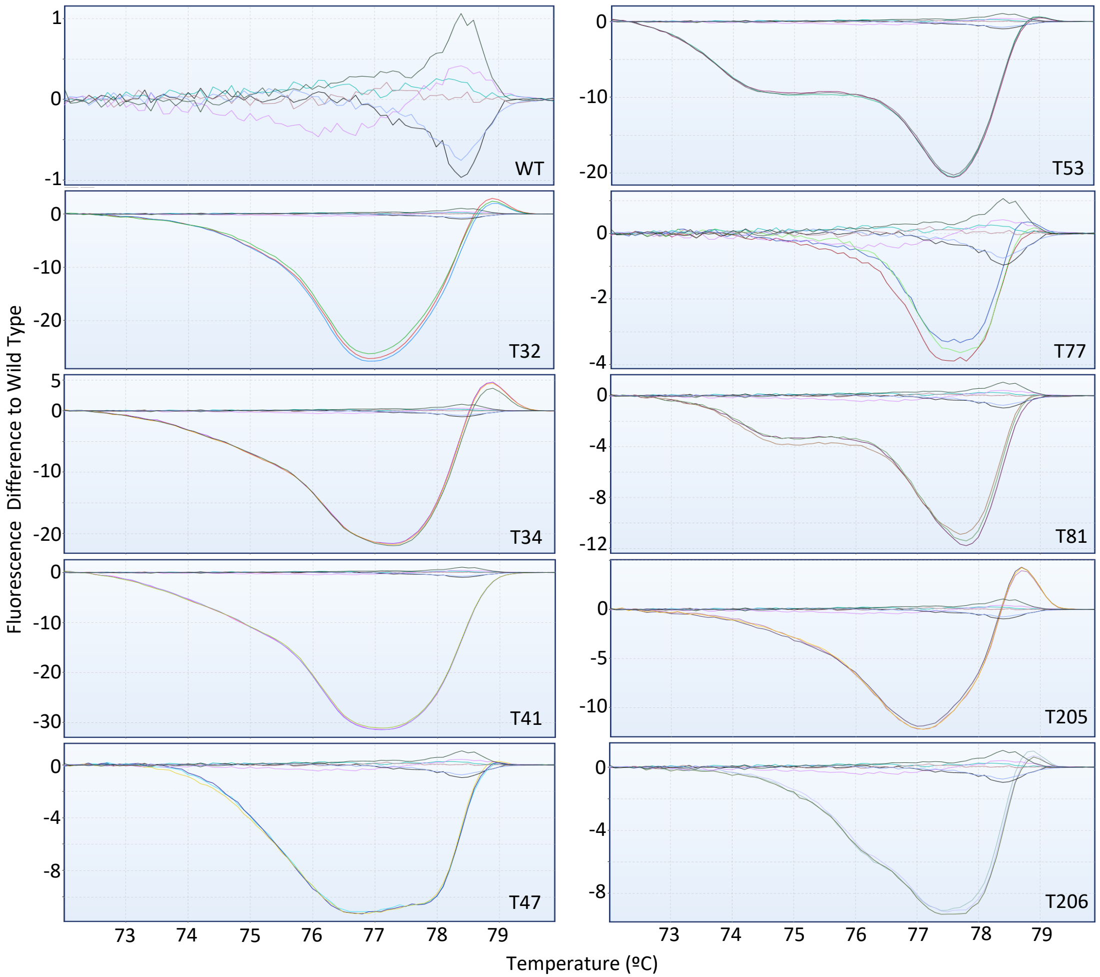

Supplementary Figure 6. High-resolution melt analysis (HRMA) fluorescence difference to WT graphs for *tgd5* lines at *TGD5* sgRNA73.

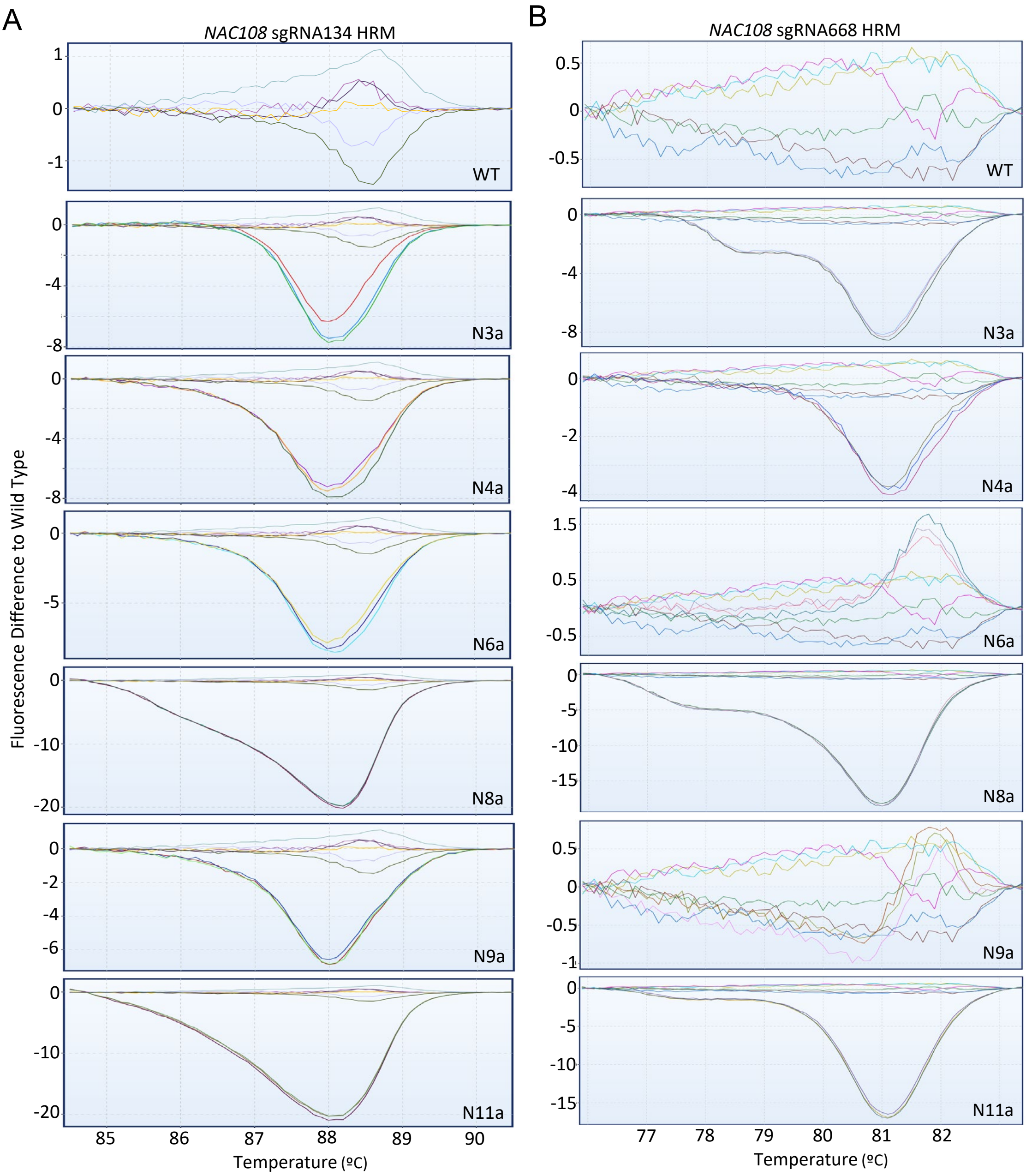

Supplementary Figure 7. High-resolution melt analysis (HRMA) fluorescence difference to WT graphs for *nac108* lines. A) *NAC108* sgRNA134 assay; B) *NAC108* sgRNA668 assay.

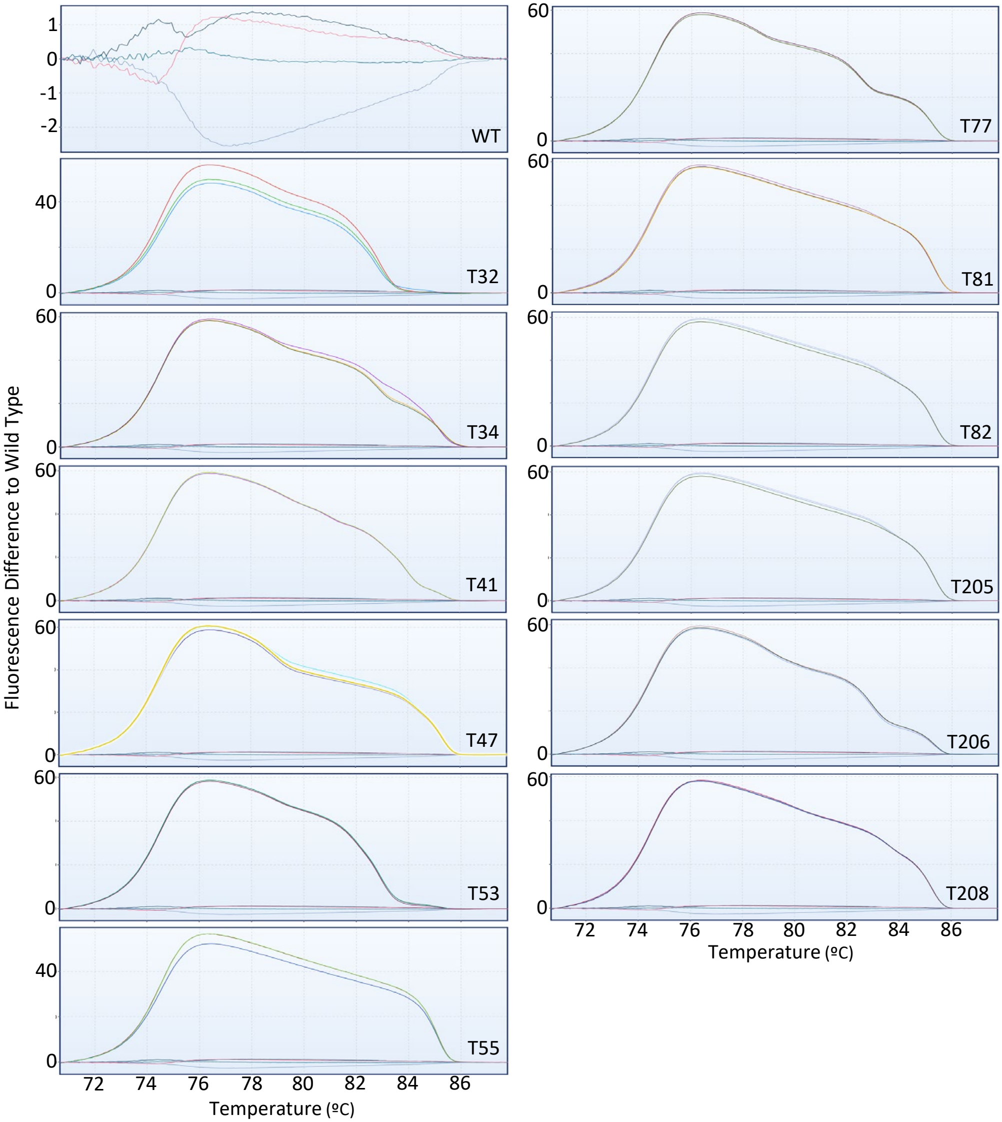

Supplementary Figure 8. High-resolution melt analysis (HRMA) fluorescence difference to WT graphs for *tgd5* lines at *TGD5* sgRNA24.

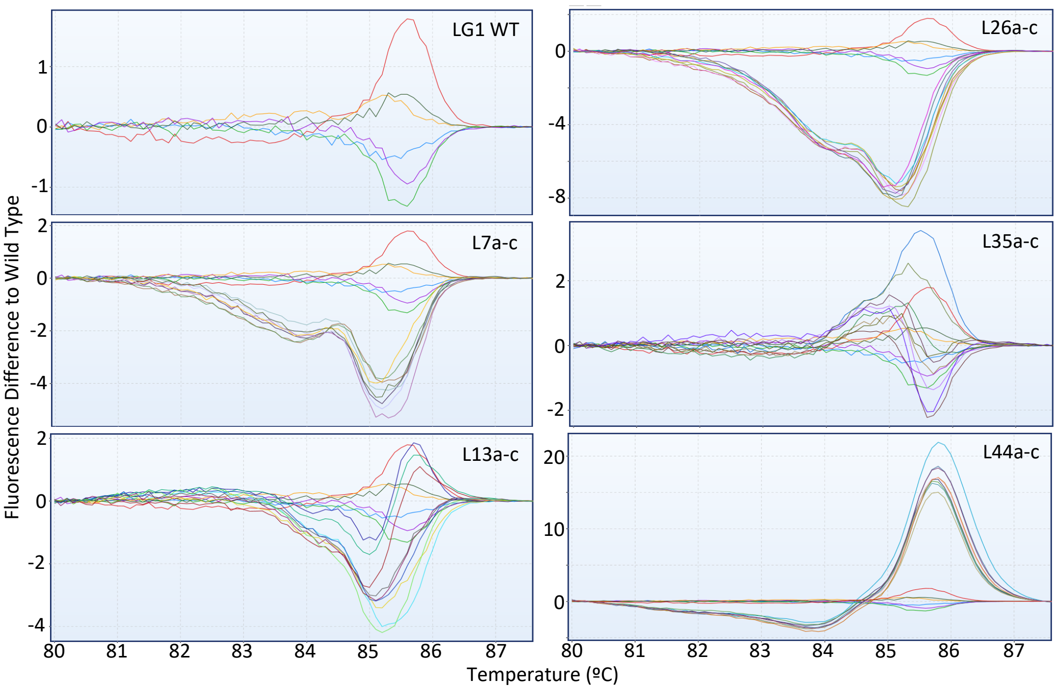

Supplementary Figure 9. High-resolution melt analysis (HRMA) fluorescence difference to WT graphs for *lg1* lines.
